# Comparative Network Analysis of Patients with Non-Small Cell Lung Cancer and Smokers for Representing Potential Therapeutic Targets

**DOI:** 10.1038/s41598-017-14195-1

**Published:** 2017-10-23

**Authors:** Mehrdad Pazhouhandeh, Fatemeh Samiee, Tahereh Boniadi, Abbas Fadaei Khedmat, Ensieh Vahedi, Mahsa Mirdamadi, Naseh Sigari, Seyed Davar Siadat, Farzam Vaziri, Abolfazl Fateh, Faezeh Ajorloo, Elham Tafsiri, Mostafa Ghanei, Fereidoun Mahboudi, Fatemeh Rahimi Jamnani

**Affiliations:** 1 Human Antibody Lab, Innovation Center, Pasteur Institute of Iran, Tehran, Iran; 2 Department of Microbial Biotechnology, Islamic Azad University, Pharmaceutical Sciences Branch, Tehran, Iran; 3 Department of Pulmonology, Shahid Beheshti University of Medical Sciences, Tehran, Iran; 4 Chemical Injuries Research Center, Baqiyatallah University of Medical Sciences, Tehran, Iran; 5 Rajaie Cardiovascular Medical and Research Center, Iran University of Medical Sciences, Tehran, Iran; 6 Internal Medicine Department, Medical Faculty, Kurdistan University of Medical Sciences, Sanandaj, Iran; 7 Microbiology Research Center, Department of Mycobacteriology and Pulmonary Research Pasteur Institute of Iran, Tehran, Iran; 8 Department of Biology, Faculty of Science, Islamic Azad University, East Tehran Branch, Tehran, Iran; 9 Molecular Medicine Department, Biotechnology Research Center, Pasteur Institute of Iran, Tehran, Iran; 10 Biotechnology Research Center, Pasteur Institute of Iran, Tehran, Iran

## Abstract

Cigarette smoking is the leading cause of lung cancer worldwide. In this study, we evaluated the serum autoantibody (AAb) repertoires of non-small cell lung cancer (NSCLC) patients and smokers (SM), leading to the identification of overactivated pathways and hubs involved in the pathogenesis of NSCLC. Surface- and solution-phase biopanning were performed on immunoglobulin G purified from the sera of NSCLC and SM groups. In total, 20 NSCLC- and 12 SM-specific peptides were detected, which were used to generate NSCLC and SM protein datasets. NSCLC- and SM-related proteins were visualized using STRING and Gephi, and their modules were analyzed using Enrichr. By integrating the overrepresented pathways such as pathways in cancer, epithelial growth factor receptor, c-Met, interleukin-4 (IL-4) and IL-6 signaling pathways, along with a set of proteins (e.g. phospholipase D (PLD), IL-4 receptor, IL-17 receptor, laminins, collagens, and mucins) into the PLD pathway and inflammatory cytokines network as the most critical events in both groups, two super networks were made to elucidate new aspects of NSCLC pathogenesis and to determine the influence of cigarette smoking on tumour formation. Taken together, assessment of the AAb repertoires using a systems biology approach can delineate the hidden events involved in various disorders.

## Introduction

Lung cancer is recognized as a fatal malignancy, accounting for more than a quarter of all cancer deaths in the United States and 1.6 million deaths every year worldwide^1,2^. Approximately 85% of all new cases of lung cancer are classified as non-small cell lung cancer (NSCLC), including adenocarcinoma, squamous cell carcinoma and large-cell carcinoma^2^. Despite an overall increase in the rate of survival for most cancers, 5-year survival for lung cancer has not improved significantly^1^. On the other hand, smoking is a primary risk factor for lung cancer, and nearly 14–25% of lifetime smokers (SM) are expected to develop lung cancer^2^. Accordingly, the carcinogenic effects of tobacco smoke through deregulation of various cellular pathways and DNA adduct formation have been extensively studied in the past decades^3^.

The low success rate of lung cancer treatment originates from delayed diagnosis, which reduces the chance of total tumour excision via surgery and acquired resistance to available medication regimens^4,5^. Imaging methods are usually applied in the diagnosis of lung cancer. However, the high costs of these methods, radiation exposure, and false positive or negative results, restrict their applications as screening tests^2,4^. Moreover, resistance to conventional chemotherapeutics, such as platinum agents, antitubulars, topoisomerase II inhibitors, and antimetabolites, is quite common among NSCLC patients who eventually develop resistance to chemotherapeutics^5^.

Autoantibody (AAb) production, as a reaction of the immune system to tumour antigens, is an inevitable phenomenon in cancers^6^. Since AAb production occurs during the early stages of disease, it may serve as a functional tool for NSCLC diagnosis^4,7^. Therefore, detection of AAbs in NSCLC patients has been the subject of a few investigations to determine the AAb repertoires and identify AAbs against particular proteins^4,7–14^. Nevertheless, for comparison of the AAb repertoires and identification of common autoantigens, limited research has been performed on the sera of NSCLC patients and SM.

In contrast to studies assessing lung cancer via bioinformatics tools^15–19^, a few studies have focused on smoking with a systems biology approach; for instance, expression-based network analysis and assessment of protein-protein interactions have revealed changes in human blood^20^, human buccal mucosa^21^, and mouse lung samples^22^ due to cigarette exposure. For exhibiting the direct correlation between smoking and lung cancer, Ying Liu *et al*. analysed three protein expression datasets of lung adenocarcinoma and divided them into two subcategories of SM and non-SM. The results revealed Ras signalling pathway (SP) and proteoglycans in cancer as the key pathways in lung cancer development in SM^23^. Furthermore, Wang *et al*. constructed a graphical model to assess gross network alterations during transition from normal to cancer tissue in SM. The findings demonstrated a gain of epithelial growth factor receptor (EGFR) and platelet-derived growth factor receptor A (PDGFRA) modules in addition to a loss of interleukin-6 (IL-6) module in lung cancer^24^.

In the present study, we carried out biopanning with a phage-displayed random peptide library on immunoglobulin G (IgG) purified from the sera of NSCLC patients and SM to detect peptides based on their AAbs. NSCLC- and SM-specific proteins, predicted from the detected peptides, were investigated with systems biology tools to demonstrate their involvement as hubs in significant pathways driving lung cancer pathogenesis. Furthermore, they were used to construct two super networks for clarifying multidimensional contribution of smoking to NSCLC.

## Results

### Peptide library enrichment led to the generation of NSCLC and SM protein datasets

**Figure 1:**
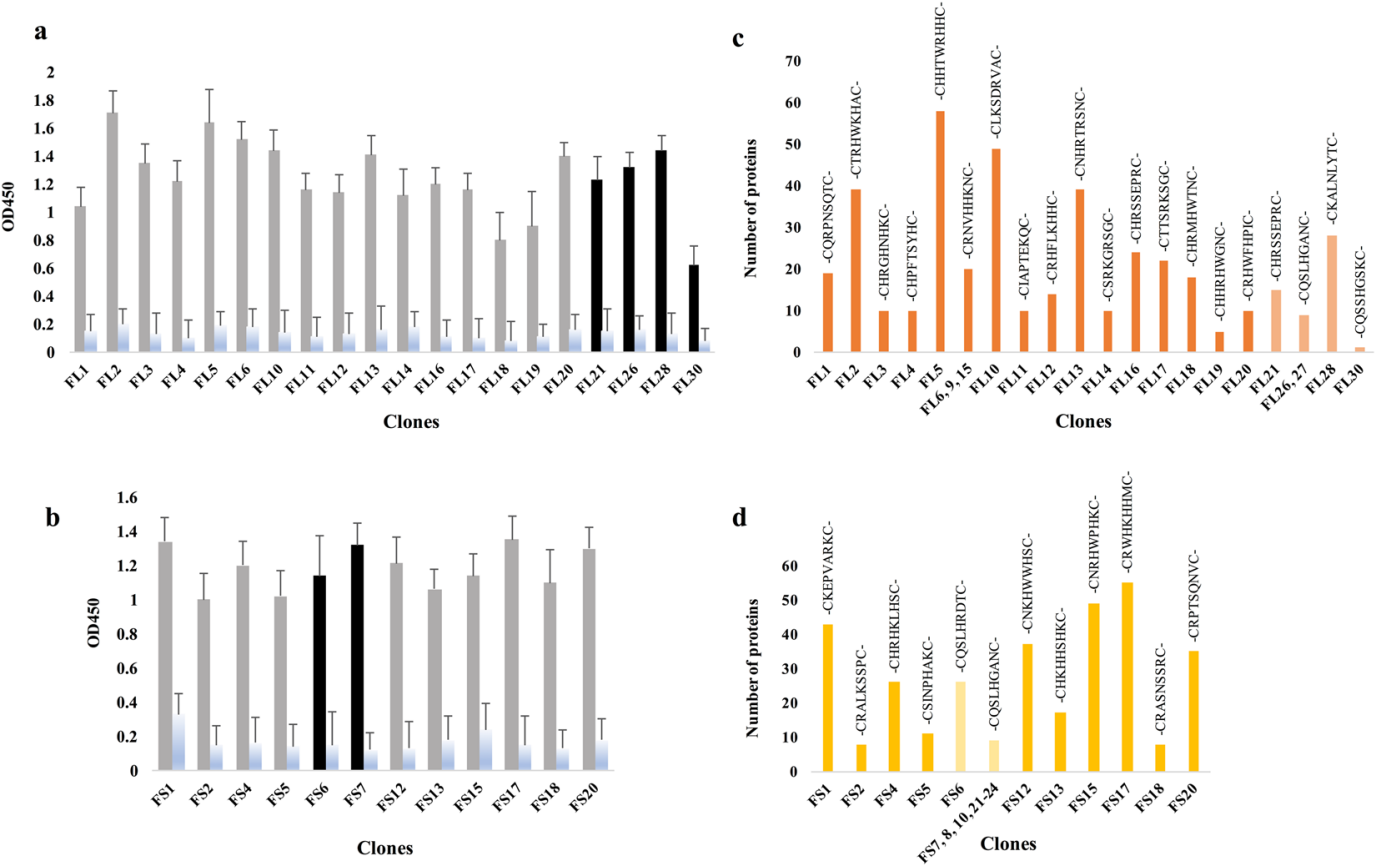
Monoclonal phage ELISA and number of proteins predicted from each selected peptide. (**a**) Randomly selected NSCLC and (**b**) SM clones were screened by monoclonal phage ELISA; intensities are reported as mean ± SD. Columns 1–10 (grey) and 11–20 (black) belong to clones from SU and SL biopanning with the highest intensities, respectively, and the blue columns indicate the controls (bovine serum albumin (BSA)). Peptides identified with the purified IgG of (**c**) NSCLC patients (orange) and (**d**) SM (yellow) are plotted against the number of human proteins which matched to each sequence after blasting with a cut-off of 18.5 and manual deletion of unrelated entities. Mild orange (**c**) and (**d**) mild yellow indicate the peptides obtained from SL biopanning.

To assess the binding of NSCLC and SM IgG to phage pools after three consecutive rounds of surface- (SU) and solution-phase (SL) biopanning, all the input and output phages were screened via polyclonal phage enzyme-linked immunosorbent assay (ELISA). The highest signals were observed in the third round of SU and SL biopanning (data not shown). Based on monoclonal phage ELISA on random clones obtained from the third round of biopanning, 30 NSCLC (SU, FL_U_1-20; SL, FL_L_21-30) and 15 SM (SU, FS_U_1-5 and FS_U_11-20; SL, FS_L_6-10 and FS_L_21-25) clones were selected, which showed the highest intensities in comparison to the controls (Fig. 1a and b). Among the designated phage clones, three FL_L_ and five FS_U_ clones failed to produce meaningful sequences. In addition, two NSCLC-specific peptides (FL_U_7 and FL_U_8) containing sequences with three consecutive histidine residues, and one SM-specific peptide (FS_L_25) due to its similarity to another peptide sequence in this group, were omitted. Notably, among NSCLC-specific clones, FL_U_6, FL_U_9, and FL_U_15 (-CRNVHHKNC-), FL_L_22 and FL_L_29 (-CTRHWPHHC-), in addition to FL_L_26 and FL_L_27 (-CQSLHGANC-), showed similar sequences (Fig. 1c). Additionally, clones FS_L_7, FS_L_8, FS_L_10, and FS_L_21-24 showed an identical amino acid composition (-CQSLHGANC-) to clones FL_L_26 and FL_L_27 (Fig. 1d).

Evaluation of the selected peptides in the MimoDB demonstrated that 21 NSCLC- and 13 SM-specific peptides, were true target binders. Blasting peptides FL_U_22, FL_U_29, and FS_L_9 (score ≥18.5) followed by an assessment in the UniProt database and review of the literature, did not present major proteins; accordingly, these peptides were deleted from the final peptide lists. However, blasting the remaining peptide sequences led to the prediction of 531 NSCLC and 346 SM proteins with 50 common proteins (Supplementary dataset). Figure 1c and d show the sequence and quantity of proteins predicted from each peptide. Furthermore, the list of proteins found in more than one clone is presented in Table S1. Peptides FL_U_19, FL_L_26, FL_L_27, and FL_L_30 accounted for the lowest number of NSCLC-related proteins (<10), while peptides FL_U_5 and FL_U_10 represented the highest number of NSCLC-related proteins (>40). Moreover, peptides FS_U_1, FS_U_15 and FS_U_17 were individually related to more than 40 proteins whereas a group of SM-specific peptides (FS_U_2 and FS_U_18, and FS_L_7, FS_L_8, FS_L_10, and FS_L_21-24) accounted for less than 10 proteins in the SM protein dataset. Markedly, retinoic acid-induced protein 1 (RAI1), dermatan-sulfate epimerase (DSE), and protein kinase C delta (PRKCD) were proteins predicted from more than one peptide in both NSCLC and SM groups.

### Protein complexes in the NSCLC and SM groups

Gene set enrichment by Consensus PathDB (CPDB) revealed multiple physically associated protein complexes, with at least two members in the NSCLC or SM protein dataset. Proline-rich tyrosine kinase 2 (PYK2)/Src homology region 2-containing protein tyrosine phosphatase 2 (SHP2) complex (two out of two components; 2/2), ADP-ribosylation factor 6 (ARF6)/Guanosine triphosphate (GTP)/Nucleoside diphosphate kinase A (NME1)/T-lymphoma invasion and metastasis-inducing protein 1 (Tiam1) complex (2/3), proto-oncogene tyrosine-protein kinase Src (SRC)-PRKCD-CUB domain-containing protein 1 (CDCP1) complex (2/3), and Janus kinase 1 (JAK1): Interleukin-13 receptor subunit alpha-1 (IL-13RA-1): Interleukin-4 receptor alpha (IL-4RA): Tyrosine kinase 2 (TYK2) complex (2/4) were among the most significant complexes in the NSCLC group (*p*-value < 0.05). APC-stimulated guanine nucleotide exchange factor 2 (ASEF2)/Adenomatous polyposis coli (APC) complex (2/2), Early elongation complex with hyperphosphorylated RNA polymerase II C-terminal domain (Pol II CTD) (4/4), Positive transcription elongation factor (P-TEFb) complex (2/3), and Tankyrin 1-tankyrin 2-telomeric repeat binding factor (TRF1) complex (2/3) were the most significant complexes in the SM group (*p*-value < 0.05). Histone deacetylase 2 (HDAC2) and lysine-specific demethylase 1 (LSD1) complexes were common in the datasets. Table S2 shows the overlapped components and *p*-value of each complex.

### Primary classification of all proteins via Pathway Studio®

**Figure 2:**
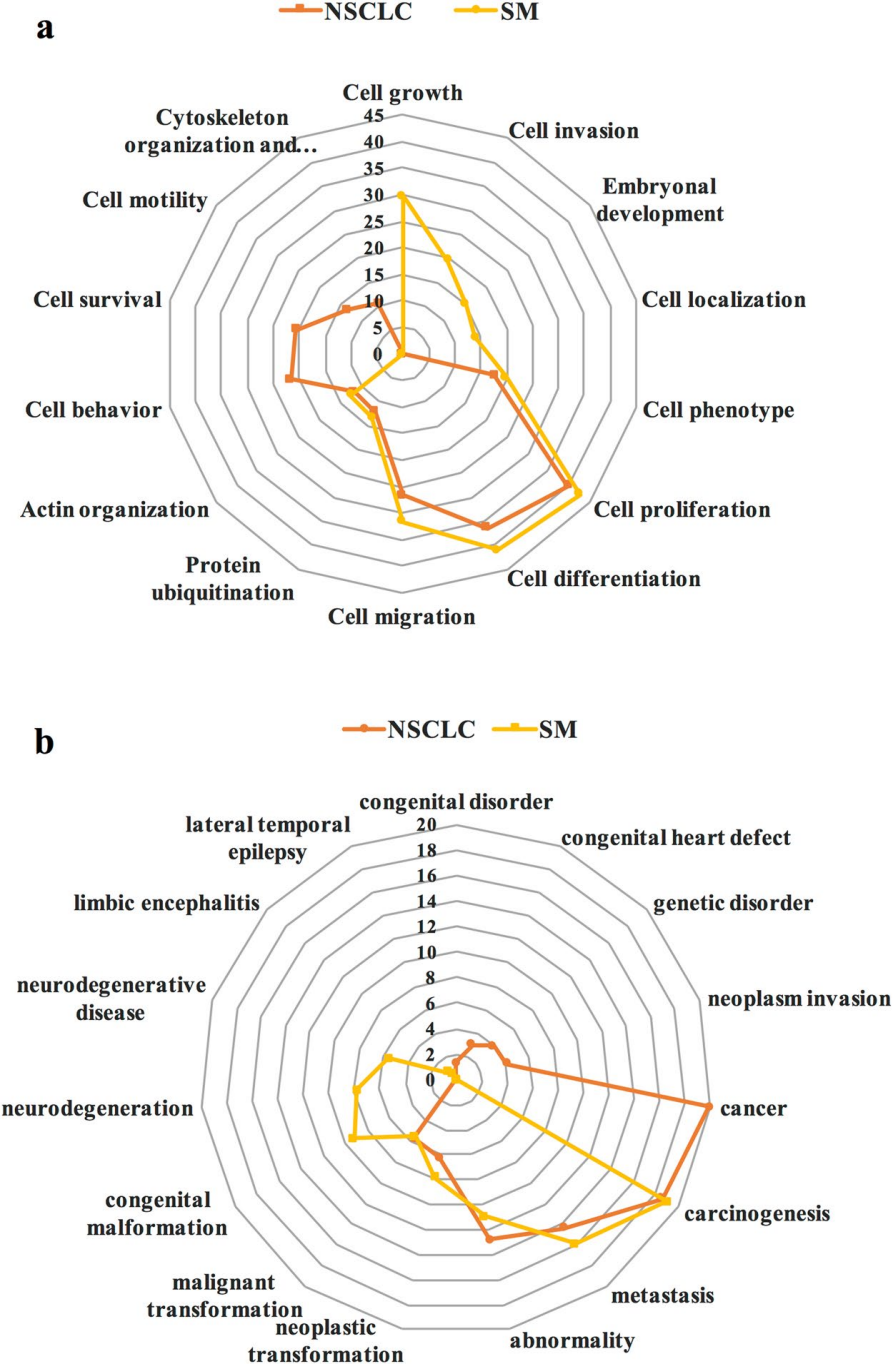
Top NSCLC- and SM-related cell processes and diseases based on Pathway Studio®. (**a**) The most significant cell processes. (**b**) The most significant diseases. Significant cell processes and diseases associated with the NSCLC (orange) and SM (yellow) groups were selected based on the network analysis function in Pathway Studio®. They are reported as the percentage of the number of proteins involved in the cell process or disease, to the total number of proteins in the related dataset (NSCLC or SM; 100 × n _involved proteins_/n _total protein in dataset_).

Network analysis of NSCLC and SM protein datasets using Pathway Studio® presented a similar pattern in some cell processes, including proliferation (40%, *p*-value: 1.7E-13; and 42%, *p*-value: 7.1E-15, respectively), differentiation (36%, *p*-value: 3.7E-23; and 41%, *p*-value: 2.9E-26, respectively), and migration (27%, *p*-value: 5.0E-19; and 32%, *p*-value: 2.9E-23, respectively) as the top three processes associated with the highest proteins count (Fig. 2a). Although cell growth seemed to be an important phenomenon in the SM group, lack of cell survival arm was witnessed.

Diseases analysis demonstrated cancer (20%, *p*-value: 3.1E-6), carcinogenesis (19%, *p*-value: 2.1E-19), metastasis (14%, *p*-value: 3.2E-10), abnormality (13%, *p*-value: 5.6E-10), neoplastic transformation (6%, *p*-value: 6.8E-7), malignant transformation (6%, *p*-value: 4.3E-7), and neoplasm invasion (4%, *p*-value: 4.1E-5), as the most important diseases linked to the NSCLC group (Fig. 2b). Moreover, carcinogenesis (*p*-value: 1.9E-14) was a major event, involving 19% of SM proteins. Other events in this group included metastasis (16%, *p*-value: 7.3E-10), abnormality (11%, *p*-value: 7.0E-5), congenital malformation (9%, *p*-value: 1.8E-8), neoplastic transformation (8%, *p*-value: 5.0E-8), neurodegeneration (8%, *p*-value: 1.5E-5), malignant transformation (5%, *p*-value: 5.9E-5), and neurodegenerative disease (5%, *p*-value: 6.3E-5). Although carcinogenesis, metastasis, and abnormality owned similar shares in both groups, cancer state and neurodegenerative disease were major disorders which were specifically associated with the NSCLC and SM groups, respectively. The percentage indicates the ratio of involved proteins to the total number of proteins in each dataset.

### Gene Ontology (GO) and pathway enrichment analysis of NSCLC and SM protein datasets

**Figure 3:**
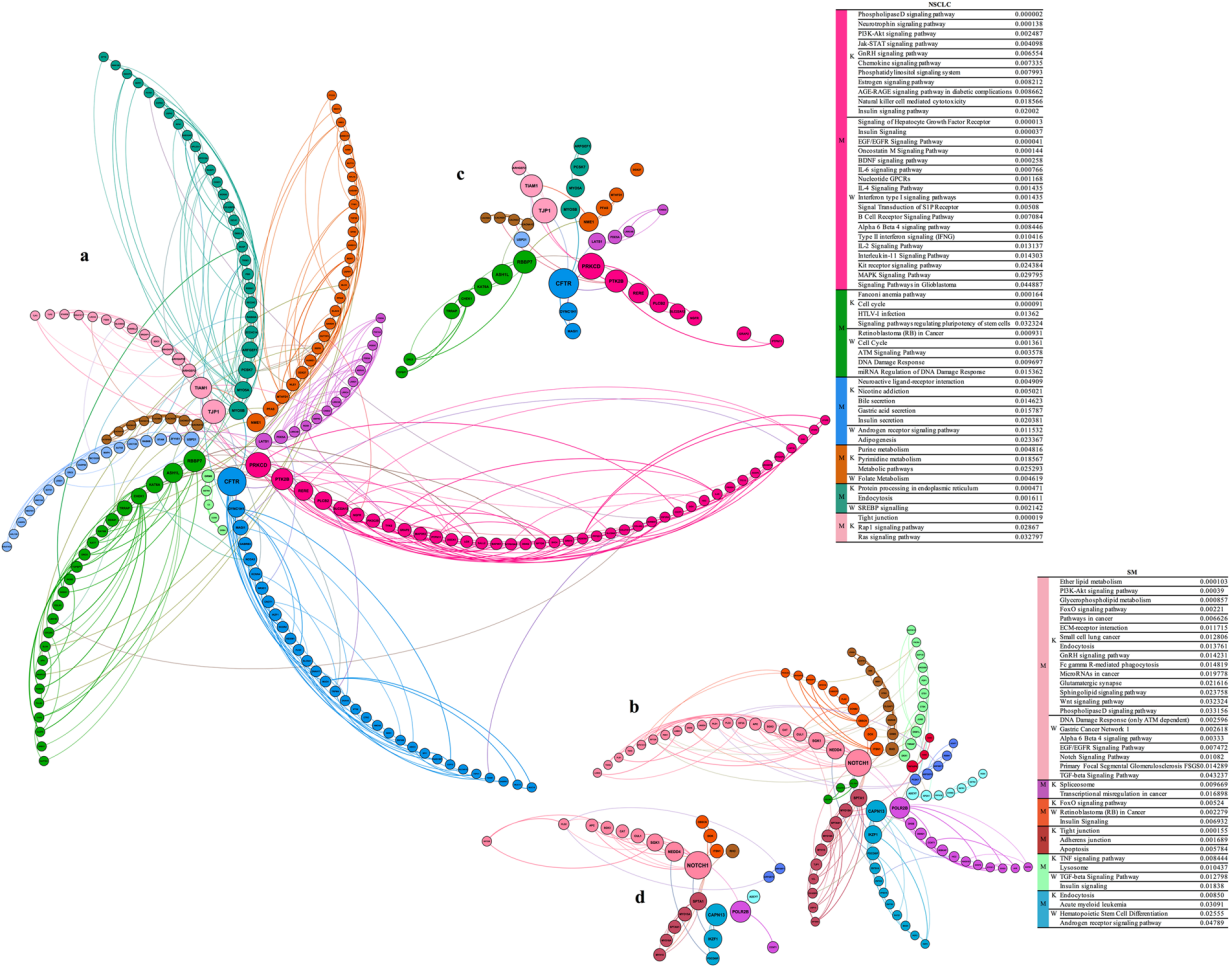
Network visualization, modularity and pathway analysis of NSCLC and SM. The node size demonstrates the betweenness centrality and the edge thickness shows the combined score of STRING. Pathway enrichment analysis of the top six modules (M) of (**a**) NSCLC and (**b**) SM was performed using Enrichr (based on the KEGG and WikiPathways databases). (**c**) NSCLC- and (**d**) SM-specific hubs are also displayed, respectively.

The NSCLC network constructed via Gephi contained 316 nodes and 513 edges, while the SM network consisted of 179 nodes and 255 edges (Fig. 3a). GO enrichment of the top six NSCLC- and SM-related modules by Metascape exhibited terms such as transmembrane receptor protein tyrosine kinase SP (GO:0007169), phospholipid metabolic process (GO:0006644), intrinsic apoptotic SP in response to oxidative stress (GO:0008631), collagen trimer (GO:0005581), DNA metabolic process (GO:0006259), signal transduction by p53 class mediator (GO:0072331), chromatin remodelling (GO:0006338), integrin-mediated SP (GO:0007229), cellular response to organic cyclic compound (GO:0071407), O-glycan processing (GO:0016266), regulation of GTPase activity (GO:0043087), phospholipid binding (GO:0005543), and apical junction complex (GO:0043296) in the NSCLC group, as well as regulation of cell migration (GO:0030334), inositol lipid-mediated signalling (GO:0048017), regulation of canonical Wnt SP (GO:0060828), DNA repair (GO:0006281), mitotic cell cycle process (GO:1903047), regulation of small GTPase-mediated signal transduction (GO:0051056), kinase activity (GO:0016301), calmodulin binding (GO:0005516), and toll-like receptor SP (GO:0002224) in the SM group. Furthermore, there were some terms related to cardiovascular diseases and diabetes which are highly prevalent disorders among Iranian population (as also confirmed among the participants of this study)^25,26^.

Expectedly, pathways enrichment analysis based on the KEGG and WikiPathways databases in Enrichr presented a group of important pathways in the top six modules of NSCLC, including phospholipase D (PLD) SP, phosphoinositide 3-kinase-Akt (PI3K-Akt) SP, JAK-signal transducer and activator of transcription (JAK-STAT) SP, gonadotropin-releasing hormone (GnRH) SP, chemokine SP, natural killer cell (NKC) mediated cytotoxicity, pathways in cancer, signalling of hepatocyte growth factor receptor (HGFR), EGF/EGFR SP, IL-4 SP, IL-6 SP, type II interferon signalling (IFNγ), mitogen-activated protein kinase (MAPK) SP, signalling pathways regulating pluripotency of stem cells, ataxia telangiectasia mutated (ATM) SP, Fanconi anaemia pathway, nicotine addiction, gamma-aminobutyric acid (GABAergic) synapse, and Ras SP (Fig. 3a). Similarly, critical pathways were found in the SM group, such as PI3K-Akt SP, pathways in cancer, extracellular matrix (ECM)-receptor interaction, Fc gamma R-mediated phagocytosis, microRNAs in cancer, glutamatergic synapse, Wnt SP, PLD SP, DNA damage response (DDR) only ATM dependent, alpha 6 Beta 4 SP, EGF/EGFR SP, neurogenic locus notch homolog protein (NOTCH) SP, transforming growth factor beta (TGF-β) SP, transcriptional misregulation in cancer, Forkhead box O (FoxO) SP, tight junction, and tumour necrosis factor (TNF) SP (Fig. 3b). Table S3 summarizes the information on some significant pathways identified via data mining.

### CFTR, PRKCD, PTK2B, CHEK1, and NOTCH1 as functional hubs

According to features such as betweenness centrality and degree implemented in Gephi, several NSCLC- and SM-specific hubs were identified (Table S4a). Proteins such as cystic fibrosis transmembrane conductance regulator (CFTR), PRKCD, tight junction protein ZO-1 (TJP1), PTK2B, TIAM1, nucleoside diphosphate kinase A (NME1), phospholipase C beta 2 (PLCB2), brefeldin A-inhibited guanine nucleotide-exchange protein 1 (ARFGEF1), and checkpoint kinase 1 (CHEK1) were NSCLC-specific hubs. Furthermore, NOTCH1, calpain-13 (CAPN13), DNA-directed RNA polymerase II subunit RPB2 (POLR2B), E3 ubiquitin-protein ligase NEDD4 (NEDD4), serine/threonine-protein kinase Sgk1 (SGK1), serine/threonine-protein kinase Sgk3 (SGK3), APC, PLD2, ARFGEF1, and regulatory-associated protein of mTOR (RPTOR) were highly ranked proteins in the SM group.

Assessment of NSCLC-specific hubs in the WebGestalt database demonstrated disorders such as lung neoplasms (*p*-value: 0.014), epithelial cancers (*p*-value: 0.014), lung diseases (*p*-value: 0.015), and carcinoma (*p*-value: 0.025), as well as several malignancies and disorders related to cancer. Evaluation of SM-specific hubs revealed confirmative results such as precancerous conditions (*p*-value: 0.003), substance-related disorders (*p*-value: 0.008), chromosome aberrations (*p*-value: 0.016), and stress (*p*-value: 0.021), in addition to adenocarcinoma and neoplasms.

Potential drugs, which could modulate NSCLC hubs, were determined by searching DrugBank and Reaxys® (Table S4b). Among NSCLC-specific hubs, a group of drugs was found to target PRKCD, PTK2B, and CHEK1, as indicated in several types of malignancies. In this regard, drugs such as bosutinib, dasatinib, axatinib, and lapatinib have been developed to concurrently inhibit these three kinases. Astonishingly, PTK2B inhibitors, crizotinib and mereletinib, are prescribed for patients with metastatic NSCLC showing specific molecular characteristics^27,28^.

### Ability of the selected hubs for discrimination of NSCLC, SM, and control sera

**Figure 4:**
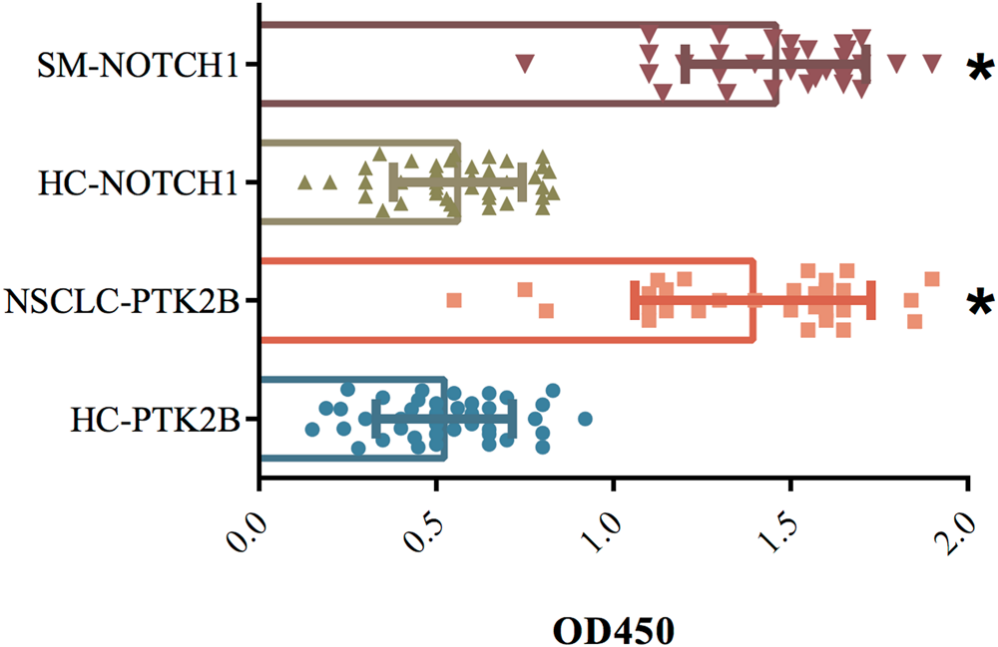
Binding assessment of the selected hubs to the NSCLC and SM sera. The results of PTK2B and NOTCH1 binding to the sera of 30 NSCLC patients (peach), 30 SM (red), and 40 age-matched healthy subjects (HC, as the controls) are presented (*p-value < 0.01).

Based on features such as betweenness centrality and degree, PTK2B and NOTCH1 were selected from the NSCLC and SM groups, respectively. The data exhibited specific binding of two hubs to the corresponding NSCLC and SM sera (*p*-value < 0.01). In contrast, they showed poor binding to the sera of age-matched healthy subjects (Fig. 4).

### The PLD SP and inflammatory cytokines network as overriding events in the NSCLC and SM groups

**Figure 5:**
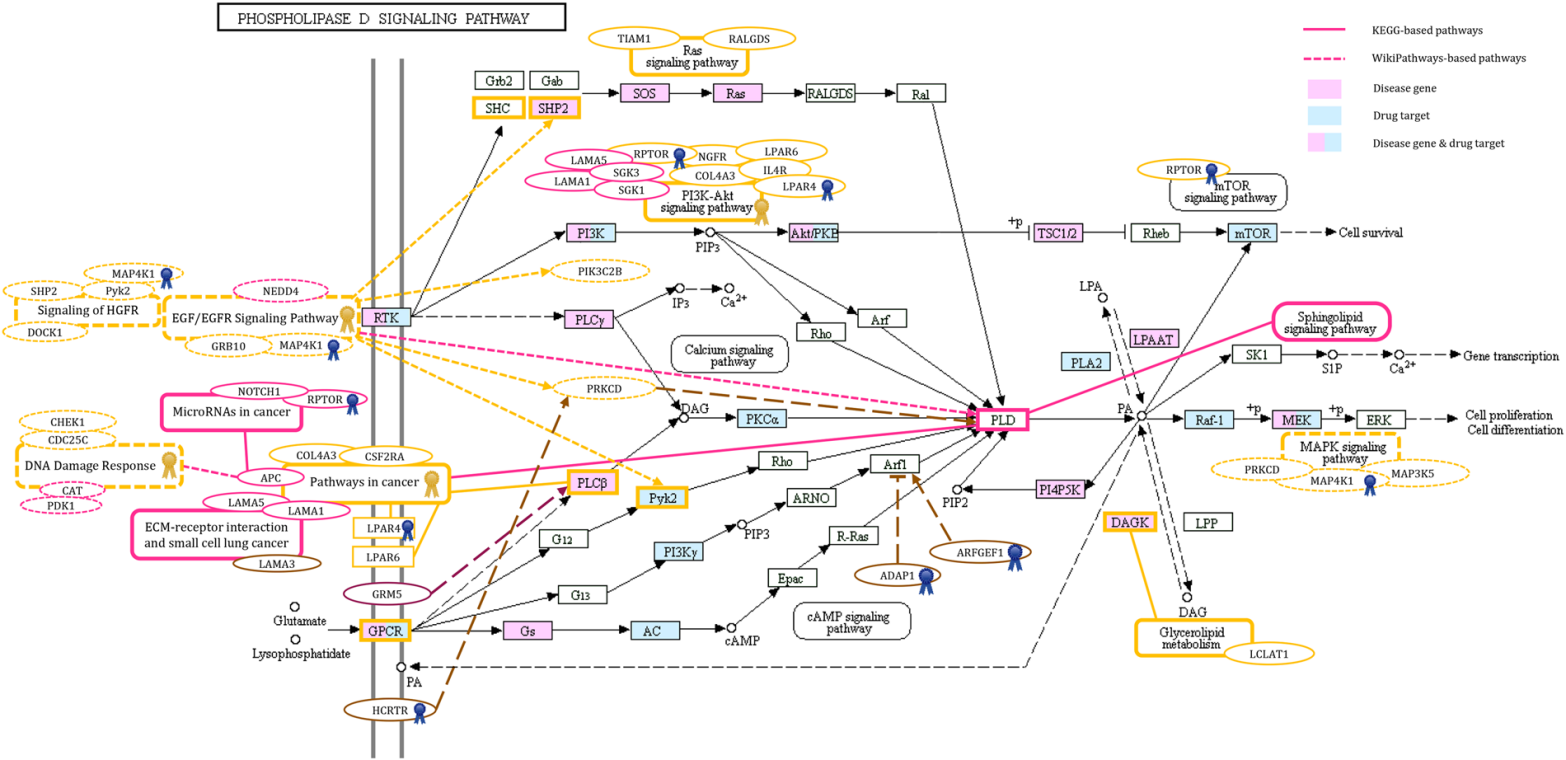
The super network constructed based on the PLD SP. The network was established based on the PLD SP in the KEGG database^30^ and was expanded by the addition of some pathways and neighbours via WikiPathways and data mining in the literature. As the identified pathways are shown in this figure, the FcεRI SP has been deleted from the PLD SP. LPAR4, LPAR6, GRM5, HCRTR1, and EGFR are the known receptors in this network. Different genes involved in the PLD SP and other related pathways are presented as coloured boxes. Yellow and pink colours indicate NSCLC- and SM-specific proteins in significant pathways found in this study, respectively. Brown and plum colours correspond to proteins from the NSCLC and SM protein datasets, respectively. Additionally, blue and yellow ribbons show common proteins and pathways between the two groups, respectively.

The PLD SP was undoubtedly the most significant NSCLC pathway. Remarkably, PLD was found among SM-specific proteins. Furthermore, phospholipid metabolic process and phospholipid binding were overrepresented GO terms identified through NSCLC-related module analysis. According to the literature review and evaluation of pathways involved in tumour formation and development, the PLD SP took centre stage in the pathogenesis of NSCLC in this study. Curation of the PLD SP derived from the KEGG database with the information attained through data-mining and Pathway Studio® led to the formation of a super network constructed with several NSCLC (e.g. PRKCD, PTK2B, PLCB2, and ARFGEF1) and SM (e.g. PLD2, ARFGEF1, and RPTOR) hubs. Among the mentioned hubs, RPTOR, a SM-specific hub, was also found in the NSCLC protein dataset. As exhibited in Fig. 5, a group of NSCLC proteins such as SHC-transforming protein 4 (SHC4), SHP2, mitogen-activated protein kinase kinase kinase kinase 1 (MAP4K1), phosphatidylinositol 4-phosphate 3-kinase C2 domain-containing subunit beta (PIK3C2B), diacylglycerol kinase theta (DGKQ), laminin subunit alpha-3 (LAMA3), arf-GAP with dual PH domain-containing protein 1 (ADAP1), ral guanine nucleotide dissociation stimulator (RALGDS), and G-protein-coupled receptors (GPCRs, including lysophosphatidic acid receptor 4 (LPAR4), lysophosphatidic acid receptor 6 (LPAR6), and orexin receptor type 1 (HCRTR1)) linked to the PLD SP, were observed in this network. Among the mentioned proteins, LPAR4, HCRTR1, and MAP4K1 were present in the SM protein dataset, as well. In addition, there were several key proteins such as NOTCH1, APC, pyruvate dehydrogenase (acetyl-transferring) kinase isozyme 1, mitochondrial (PDK1), laminin subunit alpha-1 (LAMA1), laminin subunit alpha-5 (LAMA5), and metabotropic glutamate receptor 5 (GRM5) which were only found in the SM protein dataset. Based on the KEGG and WikiPathways databases, significant SPs such as EGF/EGFR SP, HGFR SP, MAPK SP, PI3K-Akt SP, Ras SP, glycerophospholipid metabolism, glutamatergic synapse, ECM-receptor interaction, small cell lung cancer, microRNAs in cancer, DDR, and pathways in cancer identified via analysis of NSCLC and SM modules-related pathways, were also integrated in this super network. The members of this super network were linked to each other, resulting in a cascade of events. Binding of lysophosphatidic acid (LPA) to its receptors, LPAR4 and LPAR6, activates GPCR-related pathways which stimulate PLD^29,30^. Two other GPCRs, HCRTR1 activated by neuropeptide orexin and GRM5 activated by glutamate (released due to the indirect action of HIFs^31^ like HIF3A observed in the SM group), promote various pathways leading to PLD activation^32,33^. Another remarkable receptor in this network, EGFR, stimulates PLD via multiple routes containing proteins, some of which were found in this study, as well (Fig. 5)^30^. Among proteins involved in pathways leading to PLD activation, ARF1 acts as a key protein activated and inactivated by ARFGEF1 and ADAP1, respectively^34^. Several pathways such as glycerophospholipid metabolism, sphingolipid, mechanistic target of rapamycin (mTOR), and MAPK SPs, are induced through phosphatidic acid (PA) produced by the enzymatic activity of PLD^30,35,36^. Components of ECM (LAMA1, 3, and 5) induce the synthesis of PA by activation of PLD which stimulates matrix metalloproteinases (MMPs) and enhances the invasive capacity of tumor cells^37^. Among pathways connected to the PLD SP, pathways in cancer was one of the critical events observed in both NSCLC and SM groups and was linked to other significant pathways such as microRNAs in cancer, DDR, and small cell lung cancer. A set of proteins, including LPAR4, LPAR6, collagen alpha-3(IV) chain-like (COL4A3), PLCB2, and granulocyte-macrophage colony-stimulating factor receptor subunit alpha (CSF2RA) from the NSCLC protein dataset as well as APC, LAMA1, LAMA5, and PLD1 from the SM protein dataset, were involved in pathways in cancer. All NSCLC- and SM-specific proteins playing roles in the above-mentioned SPs are presented in Fig. 5.

**Figure 6:**
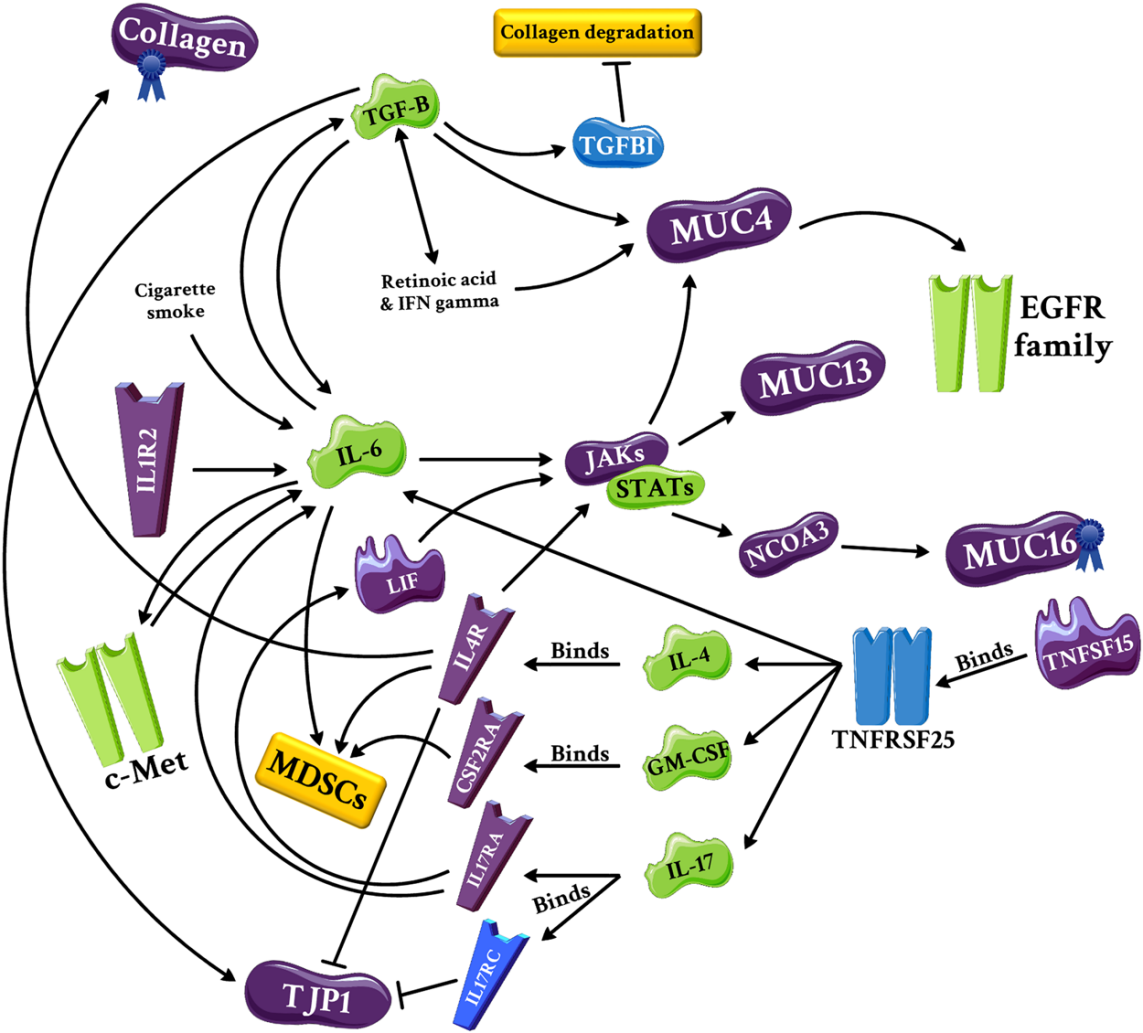
The super network constructed based on the relationship between inflammatory cytokines and tumour microenvironment. Similar to the first super network, the second network was created by proteins, particularly inflammatory cytokines, involved in significant NSCLC and SM pathways. IL-4, IL-6, IL-17, GM-CSF, and TGF-β are the most important cytokines in this network. Purple, blue, and green colours indicate presence of protein in the NSCLC protein dataset, presence of protein in the SM dataset, and unavailability of proteins in the datasets, respectively. Blue ribbons also show the presence of protein in the SM protein dataset.

The second super network was constructed to describe how cytokines alter the tumour microenvironment (e.g. ECM proteins) and how tumour recruits them to fabricate the immune system (Fig. 6). This network was originated from multiple significant pathways such as JAK-STAT SP, chemokine SP, NKC-mediated cytotoxicity, signalling of HGFR (c-Met), EGF/EGFR SP, IL-4 SP, IL-6 SP, and type II interferon signalling obtained from the NSCLC module analysis and ECM-receptor interaction, Fc gamma R-mediated phagocytosis, TGF- β SP, tight junction, and TNF SP obtained from the SM module analysis. It consisted of various proteins, including TJP1 (NSCLC hub), leukemia inhibitory factor (LIF), IL-4R, IL-1 receptor type 2 (IL-1R2), IL-17 receptor A (IL-17RA), CSF2RA, TNF ligand superfamily member 15 (TNFSF15), TYK2, PIK3C2B, nuclear receptor coactivator 3 (NCOA3), mucin-4 (MUC4), MUC13, MUC16, COL4A3, collagen alpha-1(XXVI) chain (COL26A1), and collagen alpha-1(XXVII) chain (COL27A1) which were present in the NSCLC protein dataset. SM-specific proteins such as TNF receptor superfamily member 25 (TNFRSF25), IL-17 receptor C (IL-17RC), TGF-beta-induced protein ig-h3 (TGFBI), MUC16, collagen alpha-1(III) chain (COL3A1), collagen alpha-1(XVI) chain (COL16A1), and COL27A1, were also integrated into the network. A remarkable relationship was found among the predicted proteins in this network. One of the most important pathways in the NSCLC group was IL-6 SP. IL-6 production is induced by IL-1R2^38^, IL-17^39,40^ and cigarette smoking^41^. There is a two-way interaction between IL-6 and HGF, resulting in increased invasiveness of a lung cancer cell line^42^ and an autocrine loop between IL-6 and TGF-β1 which its dysregulation causes an array of inflammatory disorders^43^. As shown in Fig. 6, IL-6 can augment the expression of MUC4 and MUC13 through JAK/STAT SP^44,45^. Production of LIF, an IL-6 family cytokine, by synovial fibroblasts is also induced by IL-17^39^. Another interleukin, IL-4, can increase the transcription of MUC4 (via JAK/STAT) and MUC16 (through JAK/STAT and NCOA3)^44,46,47^. Furthermore, IL-4 triggers M2 macrophages to produce much more collagen a3 (VI)^48^. Expression of TJP1 is also decreased by IL-4, and its distribution at the interface of adjacent cells is disrupted by IL-17 receptor C (IL-17RC)^49,50^. The major cytokine in this network, TGF-β, contributes to the modification of tumour microenvironment via MUC4 upregulation in an interdependent manner with retinoic acid and interferon type II (IFNγ)^51^. TGF-β increases TJP1 expression^52^ and inhibits collagens degradation through activation of TGFBI^53^. Locally-secreted cytokines not only modulate the structure of tumour microenvironment, but also disrupt the protective function of the immune system. TGF-β signalling plays critical roles in the inhibition of tumour-specific CD8 T cell- and NK cell-mediated cytotoxicity through regulatory T cells^54,55^. In addition, IL-4, IL-6, and granulocyte-macrophage colony-stimulating factor (GM-CSF) increase the population of myeloid-derived suppressor cells (MDSCs) which interrupt the identification of tumour antigens by T cells through induction of nitric oxide synthase and consequent nitration of T cell receptor^56^. The final link is related to the TNF family which seems to be involved in tumour suppression^57^. However, the binding of TNFSF15 to its receptor, TNFRSF25, amplifies the expression of IL-4, IL-6, GM-CSF, and IL-17^58,59^, which may explain the overactivity of these tumour-promoting cytokines in our network.

## Discussion

A combination of events such as cigarette smoking, nicotine addiction, delayed diagnosis, and resistance development during chemotherapy, has led to major concerns about lung cancer. Therefore, it can be useful to compare the AAb repertoires of NSCLC patients with SM to discover proteins involved in lung cancer pathogenesis^60,61^. According to the literature, AAbs exist in the plasma of patients several months to years before the emergence of any clinical signs, reflecting the functional immunoreactivity against tumour antigens^62^. AAbs against tumour-specific antigens generated due to antigen mutation, overexpression, or altered structure, have been recruited to discover cancer biomarkers^63^. Aside from the important role of AAbs in the discovery of tumour antigens for the development of T cell-based vaccines, they can act as therapeutic antibodies by either blocking tumour antigens or recruiting the immune system^63^. Various studies have assessed cancer-specific antibodies in different types of malignancies^63^. Similarly, several studies have examined NSCLC patients and have identified a panel of AAbs binding to rho-associated protein kinase 1, protein kinase C beta, complement factor H, c-myc, cyclin A, B1 and D1, cyclin-dependent kinase 2, MUC1, Dickkopf-related protein 1, p53, NY-ESO-1, and annexins (I and II)^8–14^. Furthermore, Yao, *et al*. introduced four combined AAb biomarkers identifying nucleolar and coiled-body phosphoprotein 1, metastasis-associated lung adenocarcinoma transcription 1, hyaluronan mediated motility receptor, and spermine oxidase for early detection of NSCLC^7^.

In the present study, classification of the predicted proteins into significant cellular processes revealed some overlapped areas as well as dissimilarities in the cellular behaviour of NSCLC and SM. Network enrichment analysis of NSCLC- and SM-related modules resulted in the identification of remarkable pathways which play key roles in cancer initiation, progression, or suppression. NSCLC-specific pathways included MAPK^64,65^, PI3K-Akt^66^, PLD^36^, Ras^67^, EGF/EGFR^68–71^, c-Met^72–74^, LPARs^75^, IL-4^76^ and IL-6^77–79^ SPs which play significant roles in tumorigenesis and cancer development. Remarkably, we found tumour suppressor pathways such as Fanconi anaemia pathway^80^ and ATM SP^81^ in the NSCLC group, which might be activated as feedbacks to the overactivated pathways in cancer cells.

Considerably, analysis of SM-related modules revealed multiple tumour-promoting pathways such as PI3K-Akt, PLD, and EGF/EGFR SPs which were commonly found in the NSCLC group. Conversely, activation of tumour suppressor pathways, including DDR and FoxO^82^ SPs as well as negative regulation of Wnt SP^83^ in the SM group, show activation of cellular defensive mechanisms against the carcinogenic compounds of cigarette smoke. Moreover, we found TGF-β and TNF SPs in this group, known as “double-edged swords”, which can promote or suppress cancers^84,85^. As the results demonstrate, carcinogenic events are strongly significant in SM and may accelerate the emergence of disorders such as cancer.

Among NSCLC-specific hubs, there are four proteins which can be considered as the key players involved in lung cancer development. The first hub is CFTR which its dysregulation has been reported in a group of cancers such as breast, gastric, prostate, lung, and colorectal cancers (CRC)^86^. Moreover, a significant correlation has been reported between CFTR downregulation and lung cancer progression and metastasis^86,87^. In contrast to studies reporting CFTR suppression in epithelial cells due to exposure to cigarette smoke, Li, *et al*. showed CFTR upregulation through nicotine-derived nitrosamine ketone (NNK), as one of the most toxic tobacco-specific carcinogens in cigarette smoke^88^. CFTR as an ABC transporter promotes protection against tobacco-specific carcinogens in cigarette smoke^88^. On the other hand, AAbs can lead to the development of lung cancer by blocking CFTR.

The second hub, PTK2B, is a part of protein complexes found in this study. This hub plays major roles in cancer cells due to its connections to critical pathways such as PLD, EGFR, and c-Met SPs^30^. It has been reported that PTK2B is upregulated in NSCLC. In fact, its higher expression and greater activity lead to the progression of NSCLC through modulating extracellular signal-regulated kinase 1/2 (ERK1/2)^89^. Furthermore, wound-induced activation of PTK2B stimulates EGFR activation in the epithelial cells of lung lesions^90^. PTK2B was also detected as a sunitinib-specific target in NSCLC patients with kirsten rat sarcoma viral oncogene (KRAS) mutations^91^. Likewise, prescription of crizotinib and merelotinib in NSCLC patients underscores the importance of PTK2B in the pathogenesis of lung cancer. KRAS-activated PRKCD promotes growth, invasion, and migration of cancer cells, through MAPK signalling in NSCLC cell lines (and mouse model), whereas PRKCD activation in cells without active KRAS leads to tumour suppression^92^. In this regard, the PRKCD inhibitor, rottlerin, can effectively potentiate chemotherapy-induced apoptosis, particularly if it is used concomitantly with trastuzumab^93^. Liu, *et al*. found that CHEK1 overexpression is associated with poor overall survival in NSCLC patients, and inhibition of CHEK1 by miR-195 suppresses tumour cell growth, migration, and invasion^94^. In this regard, Xiaojie, *et al*. revealed that miR-195 synergizes with microtubule-targeting agents (eribulin and paclitaxel) via CHEK1 regulation, leading to the inhibition of NSCLC cells growth^95^. In agreement with other studies, we confirmed the involvement of CFTR, PTK2B, PRKCD, and CHEK1 in NSCLC pathogenesis.

In terms of major cancer-related proteins among SM-specific hubs, NOTCH1, ARFGEF1, PLD2, and RPTOR were strongly aligned with hallmarks of NSCLC. NOTCH1 contributes to cell proliferation, invasion, and chemoresistance. Its involvement in tumor progression and prognosis of NSCLC has been demonstrated in different studies^96,97^. Mutations and upregulation of *Notch1* have been reported in 10% and 30% of NSCLC patients, respectively^97,98^. Konishi, *et al*. showed that suppression of NOTCH signaling inhibits the growth of NSCLC^99^. Furthermore, NOTCH signaling can crosstalk with other SPs such as Wnt and TGF-β driving cancer development^98^. Therefore, targeting NOTCH signaling by therapeutic agents like antibodies which can inhibit delta-like protein 4 (Dll4) binding to NOTCH1, may be advantageous for NSCLC patients^96,98^. In this regard, a humanized anti-Dll4 antibody, demcizumab, is currently under evaluation in three Phase 1b studies on pancreatic cancer, CRC, and NSCLC^98^.

As illustrated in the results section, three interconnected proteins, ARFGEF1, PLD2, and RPTOR, were successively activated, stimulating the cancer driver mTOR SP. Intriguingly, mutations in ARFGEF1, an activator of ARF1, have been reported in lung cancer^100^. Among these three proteins, PLDs have been garnering attention as potential contributors to cellular signalling of tumours^101^. Surprisingly, PLD represented an important intersection for other pathways in this study, and the first super network was constructed based on this enzyme. The existence of PLD isozymes (PLD1 and PLD2) in the SM protein dataset indicates the role of these junction boxes in the initiation of tumorigenesis. PLD is activated through a variety of signals such as neurotransmitters, hormones, and growth factors, transducing signals into pivotal cellular events, including proliferation, secretion, respiratory burst, and actin cytoskeletal reorganization^102^. As mentioned in the results section, PLD network involves proteins such as LPAR4, LPAR6, HCRTR1, and GRM5, as well as pathways like EGF/EGFR SP, which their roles have been proven in initiation and progression of several cancers. Phosphatidic acid, the product of PLD enzymatic activity, can be also converted to LPA and form a positive loop of LPA-LPARs^30^. LPA is involved in various cancer-related processes such as proliferation, growth, and survival^75^. Even though LPAR4 and LPAR6 are newly-discovered members of LPAR family, their contributions to hepatocellular carcinoma^103^ and thyroid cancer^104^ have been demonstrated, respectively. Although HCRTR1 stimulation with high doses of orexin causes apoptosis in colon and prostate cancers^105,106^, elevated serum orexin level has not been detected in cancer patients^107^. It seems that the normal serum level of orexin is not sufficient to induce apoptosis in cancer cells. Physiologically activated HCRTR1 may activate some pathways other than apoptosis, such as the PLD SP which contributes to tumour progression^33,105^. Therefore, pharmacologically activated HCRTR1 may be promising in cancer prevention or treatment.

Hypoxia, as a common feature of tumour microenvironment, increases the transcription of glutamate receptors, promotes the activation of GRM1, 3, 4, and 5, and supports proliferation and survival of several types of tumours through MAPK and PI3K/Akt SPs^31,107^. Noteworthy, GRM4 and 8 have been reported as susceptibility genes in NSCLC^31^. One of the critical pathways in both NSCLC and SM groups is the EGFR SP. More than half of lung tumours express receptors from EGFR family and one-fifth of lung tumours carry EGFR mutations, causing proliferation, migration, and metastasis^68,71^. Despite the preliminary response of all mutated lung tumours to tyrosine kinase inhibitors, they essentially acquire resistance^68^. Activation of mTOR, a master regulator of cell growth and proliferation, is one of the most important consequences of PLD activation. Accordingly, combination therapy of mTOR antagonists with standard regimens for lung cancer has enhanced treatment efficacy in early-stage trials^108^. Similarly, the MAPK SP, triggered by both PA and PLD, is associated with P53 degradation and MMPs induction, leading to cell survival and motility, respectively^36^. Overall, the PLD super network clarifies why treatment fails in this type of lung cancer and presents new insights into NSCLC chemotherapy. Moreover, this network underscores the pivotal function of PLDs in the occurrence of lung cancer in smokers.

Another super network constructed in this study is a complicated linkage between inflammatory cytokines and tumour microenvironment. Inflammation is a mixed blessing in cancer and the T helper 2 response deteriorates the state of cancer through suppression of cellular immunity^56^. Incidentally, the significance of IL-4 and IL-6 SPs in the present study may suggest inappropriate activation of this unfavourable arm of immunity. IL-4 SP promotes metastasis by changing the microenvironment of epithelial tumours^76^. It has been reported that serum IL-6 levels in NSCLC patients (especially metastatic cases) are higher than healthy subjects^77,78^, and blockade of IL-6/STAT3 signalling results in the inhibition of lung tumour growth^109^. Besides, STAT3 is persistently activated in GPCR C5a-knockout (KO) mice, a spontaneous lung cancer developing model, and the use of an antibody against LIF leads to STAT3 inactivation in this model^110^. The IL-6 and c-Met loop causes cell invasion through MMP activation in A549 cells, as well^42,72^. Proteins such as IL-6 and c-Met, in cooperation with IL-17 and TGF-β, play various roles in the regulation of proteins such as mucins, collagens, and TJP1. It has been demonstrated that mucins such as MUC4 and MUC13 are associated with cancer development; the circulating level of MUC16 is even used to monitor patients with ovarian cancer^111–113^. Regarding collagens, their increased and decreased expressions are correlated with malignancy^114^; accordingly, collagen XXIII has been suggested as a biomarker for the diagnosis of NSCLC^115^. Another study showed that COL4A3 cleavage by MMP-9 produces tumstatin which is a circulating anti-angiogenic factor^116^. Hence, AAb against COL4A3 may worsen the state of cancer by preventing the generation of tumstatin. Additionally, COL27A1 is aberrantly expressed in idiopathic pulmonary fibrosis^117^, a condition associated with lung cancer^118^. Ni and colleagues found that the expression of TJP1 was decreased in NSCLC tissue in comparison to adjacent non-tumour tissue and its overexpression was correlated with better prognosis in NSCLC patients^119^. Considerably, increased MDSCs population as well as dysfunction of NKCs and CD8^+^ T cells highlight an inefficient immune system in NSCLC^120–122^. Notably, our network exhibits how IL-4, IL-6, GM-CSF, and TGF-β predispose these phenomena.

In conclusion, given the rapid emergence of lung cancer in smoker populations across the world, new aspects of NSCLC pathogenesis and effects of cigarette smoke should be addressed. Tailoring the AAb profiling with a systems biology approach provides an excellent image of the network of AAbs targeting proteins which may be key drivers of cancer-interacting SPs such as PLD, PI3K-Akt, MAPK, EGF/EGFR, c-Met, Ras, DDR, IL-4, IL-6, and TGF-β SPs in the NSCLC and SM groups. Based on the PLD SP and inflammatory cytokines network, two super networks were constructed with NSCLC- and SM-specific proteins such as PLD1/2, PRKCD, PTK2B, LPARs, RPTOR, NOTCH1, TJP1, laminins, mucins, and collagens as well as pathways found in this study. Focusing on these super networks will help to design novel therapeutic agents targeting proteins involved in the pathogenesis of lung cancer.

## Methods

### Sample collection

For obtaining a repertoire of NSCLC AAbs, 67 blood samples of untreated newly-diagnosed adenocarcinoma and squamous cell carcinoma patients (stages I, Ib, II, and IIIa) were collected from different centres, including Baqiyatallah and Masih Daneshvari hospitals^123^, Tehran, Iran, as well as Kurdistan Hospital in Sanandaj, Iran. The patients aged 22–77 years (mean, 50.7 years) were all confirmed for NSCLC via histopathological tests and were classified into different stages based on the tumour/node/metastasis scale (TNM) staging system. Furthermore, 57 subjects (age range, 20–72 years; mean, 40.0 years) were allocated to the SM group according to the complete blood cell count, blood level of C-reactive protein (≤6 mg/dL), erythrocyte sedimentation rate (≤32 mm/h), rheumatoid factor (negative), and chest X-ray imaging to evaluate their overall health. All the subjects in the SM group were current smokers with different smoking patterns, ranging from a few cigarettes per week to heavy smoking.

A control group of 92 healthy non-SM volunteers (range, 11–80 years; mean, 38.9 years) were also analysed, based on the same criteria considered for the subjects in the SM group and were enrolled in the study. All healthy participants avoided anti-inflammatory drugs for three days prior to blood collection. The demographic information was collected from the subjects, and they were interviewed for potential risk factors for cancers and autoimmune diseases, including personal and family medical history. This study was done in accordance with the Helsinki Declaration^124,125^ and was approved by the Ethics Committee of Baqiyatallah University of Medical Sciences, the Ethics Committee of Masih Daneshvari Hospital^123^, and the Ethics Committee of Kurdistan University of Medical Sciences. All participants provided written informed consent before enrollment. The characteristics of all three groups are presented in Table S5.

### IgG purification from the sera of healthy control (HC), NSCLC patients, and SM

The sera of each group (HC, NSCLC, and SM) were mixed separately and IgG purification was performed using Melon™ Gel IgG Purification Kit (Pierce, Rockford, IL) according to the manufacturer’s instructions. The purification accuracy was confirmed by reducing SDS-PAGE analysis.

### Biopanning

Using Ph.D.^TM^-C7C Phage Display Peptide Library Kit (New England Biolabs, Beverly, MA), SU and SL biopanning were carried out for three successive cycles on the purified IgG of NSCLC and SM groups. For SU biopanning, 96-well Nunc MaxiSorp flat-bottom plates were coated with IgG of HC, NSCLC and SM groups. In addition, for SL biopanning, Protein G Mag Sepharose Xtra (GE healthcare, US) was employed to expose IgG, according to the manufacturer’s instructions. In both procedures, the phages of three inputs were subtracted on HC IgG before being transferred to the wells coated with IgG (NSCLC or SM IgG) or microtubes containing IgG (NSCLC or SM IgG) according to the instructions of Ph.D.^TM^-C7C Phage Display Peptide Library Kit and Protein G Mag Sepharose Xtra).

### Polyclonal and monoclonal phage ELISA

Polyclonal phage ELISA was conducted on phages obtained from three rounds of SU and SL biopanning on NSCLC and SM IgG. The specificity of peptides displayed on 60 selected phages (30 clones from SU biopanning and 30 clones from SL biopanning) to NSCLC and SM IgG was individually assayed by monoclonal phage ELISA according to the manufacturer’s instructions (Ph.D.^TM^-C7C kit) (see Supplementary information).‌

### DNA sequencing

To extract the single-stranded phage DNAs, 20 and 10 clones obtained from SU and SL biopanning on NSCLC IgG, along with 15 and 10 clones obtained from SU and SL biopanning on SM IgG, were selected, respectively, according to the Ph.D.^TM^-C7C kit instructions. After sequencing and inference of amino acid sequences by Gene Runner version 5.0, the peptides were analysed in the Biopanning Data Bank (MimoDB) (http://immunet.cn/bdb/) to discard target-unrelated peptides^126^. Based on default parameters for the Blastp (score ≥18.5), the selected peptides were blasted against the Refseq_protein database for the *Homo sapiens* ^127^.

### Determination of protein complexes in the NSCLC and SM protein datasets

NSCLC- and SM-specific proteins obtained through blasting (score ≥18.5) and preliminary analysis of the literature and UniProt database, were assessed in CPDB (http://cpdb.molgen.mpg.de) to find significant protein complexes with at least two members in the NSCLC or SM protein dataset (*p*-value < 0.05).

### Primary evaluation of NSCLC and SM protein datasets in Pathway Studio®

Pathway Studio® 11.2.5.9 (https://mammalcedfx.pathwaystudio.com) was used to exhibit the top cellular processes and diseases in two datasets. For this purpose, the NSCLC and SM protein datasets were separately introduced to Pathway Studio® and the Summary Analysis function was used to present a quick analytical summary of the datasets.

### GO and pathway-enrichment analysis of predicted proteins

For visualizing molecular interaction networks, the NSCLC and SM protein datasets were introduced to STRING 10.0 (http://string-db.org)^128^ and the nodes (proteins) and edges (protein-protein interactions) were extracted and transferred to Gephi 0.9.1^129^. The combined scores (from STRING) were defined as the edge weights. Modularity function implemented in Gephi was used for clustering, and the nodes were coloured differentially based on their modules. To identify GO terms and pathways related to the top six NSCLC and SM modules, Metascape (http://metascape.org)^130^ and Enrichr web tools (http://amp.pharm.mssm.edu/Enrichr/)^131^ were recruited. Disease Association Analysis in the WebGestalt database (http://webgestalt.org)^132^ was performed for 25 NSCLC- and 26 SM-specific nodes (hubs) which showed the highest scores of betweenness centrality and degree. Target-based search were also implemented with DrugBank 5.0 (https://drugbank.ca)^133^ and Reaxys® (https://reaxys.com) to identify marketed drugs affecting NSCLC-specific hubs.

To present a final image of NSCLC pathogenesis in this study, two super networks were constructed by combining pathways obtained from the KEGG PATHWAY and WikiPathways databases. Considering the PLD pathway as a central event in the NSCLC group, a super network was built on the PLD SP taken from the KEGG database^30^. This super network was developed by trimming the unrelated branches and adding connections based on the literature review.

Another super network was constructed, considering the effects of inflammatory cytokines on tumour microenvironment. For this purpose, proteins involved in GO terms and pathways related to inflammation or ECM, were introduced to Pathway Studio® for generating a primary network. Using “the properties function” defined in Pathway Studio®, each network component was assessed, leading to the generation of a secondary network expanded with NSCLC key proteins. Data mining was also carried out to verify this super network with the deliberated connections.

### Assessment of the selected hubs by ELISA

PTK2B (ORIGENE) and NOTCH1 (R&D systems), as the representatives of NSCLC and SM groups, were selected for ELISA, respectively (see Supplementary information).

### Statistical analysis

The statistical analyses were carried out using GraphPad prism®. The data were stated as the mean ± SD and the statistical significance (*p*-value < 0.05) was determined by two-tailed Student’s *t*-test.

## Electronic supplementary material

Supplementary Information

Supplementary Dataset
