# Supplementary material for: Comparative Network Analysis of Patients with Non-Small Cell Lung Cancer and Smokers for Representing Potential Therapeutic Targets
Source: Sci Rep. 2017 Oct 23;7:13812. doi: 10.1038/s41598-017-14195-1 (PMC5653836; doi:10.1038/s41598-017-14195-1)
Supplement: Supplementary file 1 — Supplementary Information [file 41598_2017_14195_MOESM1_ESM.doc]

**Supplementary information**

**Comparative Network Analysis of Patients with Non-Small Cell Lung Cancer and Smokers for Representing Potential Therapeutic Targets**

Mehrdad Pazhouhandeh^1^, Fatemeh Samiee^2^, Tahereh Boniadi^2^, Abbas Fadaei Khedmat^3^, Ensieh Vahedi^3^, Mahsa Mirdamadi^4^, Naseh Sigari^5^, Seyed Davar Siadat^6,1^, Farzam Vaziri^6,1^, Abolfazl Fateh^6,1^, Faezeh Ajorloo^7^, Elham Tafsiri^8^, Mostafa Ghanei^3,1*^, Fereidoun Mahboudi^9^, Fatemeh Rahimi Jamnani^6,1*^

^1^ Innovation Center, Pasteur Institute of Iran, Tehran, Iran

^2^ Department of Microbial Biotechnology, Islamic Azad University, Pharmaceutical Sciences Branch, Tehran, Iran

^3^ Chemical Injuries Research Center, Baqiyatallah University of Medical Sciences, Tehran, Iran

^4^ Rajaie Cardiovascular Medical and Research Center, Iran University of Medical Sciences, Tehran, Iran

^5^ Internal Medicine Department, Medical Faculty, Kurdistan University of Medical Sciences, Sanandaj, Iran

^6^ Microbiology Research Center, Department of Mycobacteriology and Pulmonary Research Pasteur Institute of Iran, Tehran, Iran

^7^ Department of Biology, Faculty of Science, Islamic Azad University, East Tehran Branch, Tehran, Iran

^8^ Molecular Medicine Department, Biotechnology Research Center, Pasteur Institute of Iran, Tehran, Iran

^9^ Biotechnology Research Center, Pasteur Institute of Iran, Tehran, Iran

*Corresponding author: Fatemeh Rahimi Jamnani, PharmD, PhD, Innovation Center, Pasteur Institute of Iran, Tehran, Iran. Phone: +982166953311; Fax: +982166465132; E-mail: [Rahimi@pasteur.ac.ir](mailto:rahimi@pasteur.ac.ir), [Redogone1981@gmail.com](mailto:Redogone1981@gmail.com)

*Co-corresponding author: Mostafa Ghanei, MD, pulmonologist, Chemical Injuries Research Center, Baqiyatallah University of Medical Sciences, Tehran, Iran; E-mail: m.ghanei@bmsu.ac.ir

**Methods**

- **Poly- and monoclonal phage ELISA**

The specificity of phages displaying peptides to IgG of NSCLC and SM, were evaluated by polyclonal and monoclonal phage ELISA, according to the manufacturer's instructions (Ph.D.^TM^-C7C Kit). In brief, following blocking, incubation and several times of washing with PBST (0.5% Tween-20 in phosphate buffered saline (PBS)), the isolated phages from three rounds of biopanning mentioned as inputs and outputs were added into the wells coated with IgG of NSCLC or SM and the wells coated with BSA (negative control). After washing, HRP-conjugated anti-M13 antibody was added to the wells and the plate was incubated for 1 h at room temperature (RT) (GE Healthcare, 1:5000 in 0 .1 M NaHCO3, 5 mg/ml BSA). After adding TMB substrate, the reactions were stopped by addition of HCl solution. The optical density (OD) of each well was recorded at 450 nm by microplate spectrophotometer (Epoch, BioTek). Sixty clones from the NSCLC group (30 from SU biopanning and 30 from SL biopanning) and 60 clones from the SM group (30 from SU biopanning and 30 from SL biopanning) were randomly selected from the third round of panning and examined by monoclonal phage ELISA in a similar procedure to polyclonal phage ELISA.

- **Assessment of the selected hubs by ELISA**

Two 96-well plates were coated separately with PTK2B (ORIGENE) and goat anti-human IgG Fc antibody (100ng/well) and incubated overnight at 4 ^o^C. Goat anti-human IgG Fc antibody was used for capturing Notch1-Fc chimera protein. Following washing and blocking with skimmed milk in PBS, the sera of 30 NSCLC, 30 SM and 40 age-matched healthy subjects diluted in PBS (1 mg/ml) were added to the wells containing PTB2K and the wells which have previously been incubated with recombinant human Notch1-Fc chimera protein ^1^. After several times washing with PBST, the wells were incubated with goat anti-human IgG antibody conjugated HRP (Abcam) for 1 h at RT. Following several washings with PBST, reactions were developed by TMB and stopped by HCL. The cutoff level for positive reactions was calculated according to means plus 2 SDs of the OD_450_ readings of the healthy control.

Table S1. The list of proteins predicted from more than one clone.

|  | **The proteins** | **Number of corresponding clones** |
| --- | --- | --- |
| **NSCLC** | BTBD10, C10orf76, CASD1, IFT52, MTMR3, MYOM2, PRKCD, RAI1, SPHKAP, SRBD1, ZNF224 | 3 |
|  | BTBD11, CACNA1F, CDC42BPB, CHRDL2, DDX21, DENND6A, DHRS9, DNAH12, DSE, FLG, KAT6B, KIAA1109, MYPOP, NLGN2, PRSS45, SDC3, TACC3, TREM2, XPO4, ZDHHC14, ZNF783 | 2 |
| **SM** | RAI1 | 8 |
|  | CDC42BPB, CHRDL2, DDX21, DSE, NLGN2, TACC3, TREM2, ZDHHC14 | 7 |
|  | SYNE1 | 2 |

*Table S2. Significant protein complexes. The NSCLC and SM protein datasets were assessed for the detection of physically associated protein complexes by means of CPDB. The complexes with two or more proteins from the datasets were considered as significant complexes (p-value < 0.05).*

|  | **Protein complex** | | **Matched fraction** | ***p*-value** | **Overlapped proteins** | **All members** |
| --- | --- | --- | --- | --- | --- | --- |
| NSCLC | PYK2/SHP2 | | 2/2 | 0.000632 | PTK2B, PTPN11 | PTK2B, PTPN11 |
|  | ArgBP2a-CBL-PTK2B | | 2/3 | 0.00186 | PTK2B, SORBS2 | CBL, PTK2B, SORBS2 |
|  | ARF6/GTP/NME1/Tiam1 | | 2/3 | 0.00186 | NME1, TIAM1 | ARF6, NME1, TIAM1 |
|  | SRC-PRKCD-CDCP1 | | 2/3 | 0.00186 | CDCP1, PRKCD | CDCP1, PRKCD, SRC |
|  | ING5 | | 3/11 | 0.00224 | KAT6A, KAT6B, KAT7 | BRD1, BRPF1, BRPF3, ING5, JADE1, JADE2, JADE3, KAT6A, KAT6B, KAT7, MEAF6 |
|  | JAK1:IL-13RA-1:IL-4R alpha:TYK2 | | 2/4 | 0.00367 | IL4R, TYK2 | IL13RA1, IL4R, JAK1, TYK2 |
| SM | ASEF2/APC | | 2/2 | 0.00029 | APC, SPATA13 | APC, SPATA13 |
|  | Early elongation complex with hyperphosphorylated Pol II CTD | | 4/24 | 0.000665 | CCNT1, CCNT2, GTF2F1, POLR2B | CCNT1, CCNT2, CDK9, GTF2F1, GTF2F2, POLR2A, POLR2B, POLR2C, POLR2D, POLR2E, POLR2F, POLR2G, POLR2H, POLR2I, POLR2J, POLR2K, POLR2L, SUPT4H1, SUPT5H, NELFA, NELFE, CTDP1, NELFB, NELFCD |
|  | P-TEFb | | 2/3 | 0.00086 | CCNT1, CCNT2 | CCNT1, CCNT2, CDK9 |
|  | Tankyrin 1-tankyrin 2-TRF1 | | 2/3 | 0.00086 | TNKS, TNKS2 | TERF1, TNKS, TNKS2 |
| Mutual | HDAC2 | NSCLC | 3/17 | 0.00826 | HMG20B, RBBP7, ZMYM2 | CHD3, CHD4, GSE1, GTF2IRD1, HDAC1, HDAC2, HMG20B, KDM1A, MTA1, MTA2, PHF21A, RBBP4, RBBP7, RCOR1, SIN3A, ZMYM2, ZMYM3 |
|  |  | SM | 2/17 | 0.0334 | PHF21A, RCOR1 |  |
|  | LSD1 | NSCLC | 2/13 | 0.0411 | HMG20B, ZMYM2 | CTBP1, HDAC1, HMG20A, HMG20B, HSPA1A, KDM1A, PHF21A, PHF21B, RCOR1, RCOR3, RREB1, ZMYM2, ZNF217 |
|  |  | SM | 3/13 | 0.00123 | PHF21A, RCOR1, RCOR3 |  |

Table S3. Literature review on NSCLC and SM-related pathways. SM-specific terms are presented in aqua.

| **Pathways** | **Dysregulations** | | | |
| --- | --- | --- | --- | --- |
|  | **Lung cancer** | | **Other cancers** | |
| **DDR** | Low DNA repair capacity (DRC) for repairing tobacco carcinogen-induced DNA damage is correlated with lung cancer^2,3^. Effective DRC may be related to poorer survival in NSCLC patients undergone chemotherapy^4^. | | Impaired DDR is associated with different cancers^5^. DNA repair inhibitors can be used for cancer treatment (cancer cells require DDRs)^6,7^. | |
| **EGF/EGFR SP** | EGFR signalling activation by mutation and phosphorylation are early event in NSCLC^8,9^. All EGFR mutant lung adenocarcinomas develop resistance to TKIs^8^. MET amplification plays a role in the resistance to EGFR inhibitors^8^. | | EGFR overexpression is associated with poorer prognosis^10,11^. | |
| **GnRH SP** | GnRH receptors are overexpressed in lung cancer cells^12^. GnRH promotes self-renewal and stemness maintenance in lung cancer^13^. | | Overexpression of GnRH receptors in cancers other than lung cancer, led to the antitumor activity^14^. GnRH analogues may exert anti-angiogenic activity^14^. | |
| **IL-6 SP** | Elevated serum of IL-6 level has been identified in NSCLC patients, especially in metastatic cases^15,16^. IL-6, IL-8 and TNF-alpha exist in benign and malignant pleural effusions^17^. HGF and IL-6 levels can be considered as predictors of the aggressiveness of stage III NSCLC^18^. Blocking of the IL-6/STAT3 signalling suppresses lung tumour growth^19^. Antibody against LIF suppressed Stat3 activation and spontaneous lung adenocarcinomas^20^. | | Autocrine or paracrine production of IL-6 plays important roles in various tumour behaviours and resistance^21^. LIF negatively regulates p53 and activates AKT-mTOR signalling pathway^22^. Hypoxia is involved in regulating LIF and consequent de-differentiated cancer phenotype^23^. | |
| **MAPK SP** | ERK1/2 hyperactivation is associated with advanced and aggressive NSCLC tumours^24^. MAPK/Erk1/2 SP regulates TGF-β1-induced epithelial to mesenchymal transformation of A549 cells^25^. | | Raf-MAPK/ERK kinase-ERK pathway plays roles in many cancers^26^. Ras mutations cause the persistent activation of the Ras–Raf–MEK–ERK and tumour cell proliferation^27^. | |
| **PLD SP** | RalA and PI3K cause high activity of PLD in lung carcinoma^28^. LPA or bacterial PLD enhances lung cancer cell survival^29^. Bacterial PLD or PA increases lung cancer cell invasion^30^. | | PLD1 and/or PLD2 expression are elevated in many cancers^30^ and play remarkable roles in different aspects of cancer^30^. | |
| **Purine metabolism** | Tobacco carcinogens often target the purines in DNA^31^. | | DNA turnover in cancer cells causes the elevation in purine metabolism capacity^32^ and confers selective growth advantages^33^. | |
| **Signalling of Hepatocyte Growth Factor Receptor** | MET amplification is more common than mutation in NSCLC^34,35^. HGF stimulates cell motility and activation of the focal adhesion kinase and PYK2 in SCLC^35^. MET and HGF are associated with higher pathologic tumour stage and worse prognosis^36^. MET/HGF SP inhibitors are being evaluated in lung cancer^37^. | | Met activation might be due to the auto- or paracrine in tumors^38^. Met mutations, overexpression, deregulation in degradation, and its signalling pathway have been reported in many cancers^39,40^. Hypoxia and Wnt pathway control MET expression in colorectal cancer^36^. MET regulates different aspects of cancer^40,41^. | |
|  | | **Smoking-related terms** | | **Cancer-related terms** |
| **DDR only ATM dependent** | | As a repairing system, ATM responds to DNA double-strand breaks as well as ROS formation during hypoxia^42,43^. Activation of ATM-activated phosphatidylinositol 3-kinase-related kinases in response to cigarette smoke, activates p53 and CHEK2 ^44^. Tobacco and nicotine-free cigarettes induce DDR by ATM^45^. | | ATM signalling is frequently upregulated in cancer cells that have already evaded apoptosis by other means^46^ and contributes to metastasis and cell survival by recruiting NF-κB and AKT^47,48^. Constitutive activation of ATM/CHEK2 pathway was found in p53 mutant lung cancer cells^49^. ATM and APC mutations have been reported in lung tumours, too^50^. |
| **Glutamatergic synapse** | | Nicotine can enhance the calcium-mediated release and function of glutamate^51,52^ and alters the expression of ionotropic glutamate receptor expression^52^. | | Glutamate antagonists have exhibited anti-proliferative effects and decreased the motility and invasive growth in lung adenocarcinomas^53^. The NMDA antagonist, dizocilpine, suppresses many cancer-promoting pathways^54^. Glutamate receptor subunits are expressed in many tumors^55,56^. GRM3 and GRM5 regulate proliferation, differentiation, and self-renewal of stem cells of different origin^57^. |
| **TGF-** β**SP** | | Smoking upregulates TGF-β1 in the lung epithelial lining fluid^58^. Cigarette smoke decreases TGF-β–mediated growth inhibition, and enhances tumorigenicity^59^. Cigarette smoke dysregulates TGF-β and Wnt signalling in alveolar epithelial cells, as well^60^. | | Prior to tumour initiation, TGF-β acts as a tumour suppressor but at later stages, it aids cancer progression^61^. TGF-β1 may be correlated to oncogenesis and serves as a prognostic biomarker for NSCLC^62^. Knockdown of TGF-β receptor II resulted in cell apoptosis in lung adenocarcinoma^63^. |
| **Abbreviations**  DDR: DNA damage response, PLD: phospholipase D, and SP: signalling pathway. | | | | |

**Table S4. NSCLC- and SM-specific hubs and marketed drugs that affect them.** (a) Following network visualization with Gephi, proteins with the highest score of betweenness centrality and degree were selected. (b) NSCLC hubs were investigated in DrugBank and Reaxys® to find marketed drugs modulating them (b).

| **a.** |  | | |  | | |
| --- | --- | --- | --- | --- | --- | --- |
| No. | **NSCLC** | | | **SM** | | |
|  | ID | Betweenness centrality | Degree | ID | Betweenness centrality | Degree |
| 1 | CFTR | 8275 | 12 | NOTCH1 | 4038 | 15 |
| 2 | PRKCD | 6755 | 9 | CAPN13 | 2808 | 21 |
| 3 | TJP1 | 6191 | 11 | POLR2B | 2648 | 12 |
| 4 | RBBP7 | 5328 | 12 | NEDD4 | 2198 | 6 |
| 5 | PTK2B | 5202 | 14 | IKZF1 | 2022 | 6 |
| 6 | TIAM1 | 5061 | 10 | SPTA1 | 1817 | 8 |
| 7 | ASH1L | 4315 | 12 | SGK1 | 1767 | 12 |
| 8 | RERE | 4052 | 7 | CUL1 | 1109 | 8 |
| 9 | NME1 | 3766 | 6 | MYO15A | 1047 | 5 |
| 10 | MYO5B | 3712 | 12 | ITSN1 | 1013 | 8 |
| 11 | PLCB2 | 3532 | 9 | RHO | 976 | 4 |
| 12 | MYO5A | 3314 | 13 | DCK | 972 | 5 |
| 13 | PCSK7 | 3305 | 9 | PDCD6IP | 931 | 4 |
| 14 | DYNC1H1 | 3260 | 9 | CAT | 864 | 8 |
| 15 | KAT6A | 3184 | 8 | SGK3 | 857 | 9 |
| 16 | LATS1 | 2950 | 11 | SPTAN1 | 852 | 9 |
| 17 | ARFGEF1 | 2943 | 7 | ADCY7 | 850 | 6 |
| 18 | CHEK1 | 2567 | 10 | OBSCN | 815 | 6 |
| 19 | TRRAP | 2443 | 10 | APC | 780 | 5 |
| 20 | MAGI1 | 2397 | 5 | MYO18A | 746 | 6 |
| 21 | PFAS | 2371 | 10 | MYH15 | 746 | 6 |
| 22 | GRAP2 | 1691 | 10 | CCNT1 | 500 | 6 |
| 23 | PDE5A | 1563 | 9 | PLD2 | 406 | 6 |
| 24 | CACNA1C | 908 | 11 | ARFGEF2 | 404 | 6 |
| 25 | CNBD2 | 167 | 11 | ARFGEF1 | 250 | 6 |
|  |  |  |  | RPTOR | 141 | 7 |

| **b.** | | | | | | | | | |
| --- | --- | --- | --- | --- | --- | --- | --- | --- | --- |
| **Target** | **Drug** | **DrugBank** | **Reaxys** | **pX value** | **Target** | **Drug** | **DrugBank** | **Reaxys** | **pX value** |
| CFTR | Glyburide | Antagonist | Inhibition | 5.1 | PTK2B | Leflunomide | Antagonist |  |  |
|  | Ivacaftor | Potentiator |  |  |  | Crizotinib |  | Inhibition | 7.2 |
|  | Ibuprofen | Inhibitor |  |  |  | Bosutinib |  | Inhibition | 6.8 |
|  | Lumacaftor | Modulator |  |  |  | Mereletinib |  | Inhibition | 6.1 |
|  | Bumetanide | Antagonist |  |  |  | Lapatinib |  | Inhibition | 5.8 |
|  | Crofelemer | Antagonist |  |  |  | Pazopanib |  | Inhibition | 5.6 |
|  | Piretanide |  | Inhibition | 7.2 |  | Palbociclib |  | Inhibition | 5.3 |
|  | Furosemide |  | Inhibition | 7.1 | NME1 | Tenofovir | Substrate |  |  |
|  | Quercetin |  | Inhibition | 5.8 |  | Lamivudine | Substrate |  |  |
|  | Apigenin |  | Inhibition | 5.6 |  | Adefovir | Substrate |  |  |
|  | Kaempferol |  | Inhibition | 5.6 | CHEK1 | Bosutinib |  | Inhibition | 9.8 |
|  | Myricetin |  | Inhibition | 5.6 |  | Palbociclib |  | Inhibition | 8 |
|  | Benzbromarone |  | Inhibition | 5.4 |  | Lapatinib |  | Inhibition | 7.5 |
|  | Cyclosporine |  | Inhibition | 5.3 |  | Pazopanib |  | Inhibition | 5.5 |
| PRKCD | Bosutinib |  | Inhibition | 6.4 |  | Vandetanib |  | Inhibition | 5.5 |
|  | Fasudil |  | Inhibition | 5.6 |  | Dasatinib |  | Inhibition | 5.2 |
|  | Dasatinib |  | Inhibition | 5.1 |  | Axatinib |  | Inhibition | 5.2 |
|  | Axatinib |  | Inhibition | 5.1 |  | Fasudil |  | Inhibition | 5.1 |
|  | Lapatinib |  | Inhibition | 5.8 |  |  |  |  |  |

Table S5. Baseline characteristic of NSCLC patinets, smokers, and healthy volunteers.

| **Characteristics** | | **NSCLC patients** | | | **Smokers** | | | **Healthy volunteers** | | |
| --- | --- | --- | --- | --- | --- | --- | --- | --- | --- | --- |
|  |  | Female | Male | Total | Female | Male | Total | Female | Male | Total |
| No. of people enrolled | | 16 | 53 | 69 | 10 | 53 | 63 | 76 | 47 | 123 |
| No. of people selected | | 16 | 51 | 67 | 8 | 49 | 57 | 52 | 40 | 92 |
| No. of participants excluded from the study | RF + CXR + ESR |  |  |  |  |  |  | 3 |  | 3 |
|  | RF + ESR |  |  |  |  |  |  | 3 | 1 | 4 |
|  | CRP + ESR |  |  |  |  |  |  | 2 |  | 2 |
|  | CXR + RF |  |  |  |  |  |  |  | 1 | 1 |
|  | ESR + CXR |  |  |  |  | 1 | 1 | 1 |  | 1 |
|  | CXR |  |  |  | 1 | 1 | 2 | 4 | 1 | 5 |
|  | ESR |  |  |  |  |  |  | 5 |  | 5 |
|  | CRP |  |  |  |  |  |  |  |  |  |
|  | RF |  |  |  |  | 1 | 1 |  |  |  |
|  | Others |  | 2 | 2 | 1 | 1 | 2 | 6 | 4 | 10 |
| Mean age of selected people (range) | | 52.1 (30-77) | 49.4 (22-77) | 50.7 (22-77) | 37.2 (23-59) | 42.9 (20-72) | 40.1 (20-72) | 40.2 (20-65) | 37.4 (11-80) | 38.9 (11-80) |
| Stage of disease in the selected patients | | I, Ib, II, III, IIIa  Adenocarcinoma/ Squamous cell carcinoma | | | - | | | - | | |

**Abbreviations**

CRP: C-reactive protein, CXR: Chest X-ray, ESR: Erythrocyte sedimentation rate, and RF: Rheumatoid factor.

**References:**

1 Jamnani, F. R. *et al.* Targeting high affinity and epitope-distinct oligoclonal nanobodies to HER2 over-expressing tumor cells. *Exp Cell Res* **318**, 1112-1124, doi:10.1016/j.yexcr.2012.03.004 (2012).

2 Wei, Q. *et al.* Repair of Tobacco Carcinogen-Induced DNA Adducts and Lung Cancer Risk: a Molecular Epidemiologic Study. *J Natl Cancer Inst* **92**, 1764-1772, doi:10.1093/jnci/92.21.1764 (2000).

3 Wei, Q., Cheng, L., Hong, W. K. & Spitz, M. R. Reduced DNA Repair Capacity in Lung Cancer Patients. *Cancer Res* **56**, 4103-4107 (1996).

4 Bosken, C. H., Wei, Q., Amos, C. I. & Spitz, M. R. An Analysis of DNA Repair as a Determinant of Survival in Patients With Non-Small-Cell Lung Cancer. *J Natl Cancer Inst* **94**, 1091-1099, doi:10.1093/jnci/94.14.1091 (2002).

5 Lord, C. J. & Ashworth, A. The DNA damage response and cancer therapy. *Nature* **481**, 287-294, doi:10.1038/nature10760 (2012).

6 Shaheen, M., Allen, C., Nickoloff, J. A. & Hromas, R. Synthetic lethality: exploiting the addiction of cancer to DNA repair. *Blood* **117**, 6074-6082, doi:10.1182/blood-2011-01-313734 (2011).

7 Helleday, T., Petermann, E., Lundin, C., Hodgson, B. & Sharma, R. A. DNA repair pathways as targets for cancer therapy. *Nat Rev Cancer* **8**, 193-204, doi:10.1038/nrc2342 (2008).

8 Siegelin, M. D. & Borczuk, A. C. Epidermal growth factor receptor mutations in lung adenocarcinoma. *Lab Invest* **94**, 129-137, doi:10.1038/labinvest.2013.147 (2014).

9 Cortas, T. *et al.* Activation state EGFR and STAT-3 as prognostic markers in resected non-small cell lung cancer. *Lung cancer* **55**, 349-355 (2007).

10 Mitsudomi, T. & Yatabe, Y. Epidermal growth factor receptor in relation to tumor development: EGFR gene and cancer. *The FEBS journal* **277**, 301-308, doi:10.1111/j.1742-4658.2009.07448.x (2010).

11 Normanno, N. *et al.* Epidermal growth factor receptor (EGFR) signaling in cancer. *Gene* **366**, 2-16, doi:10.1016/j.gene.2005.10.018 (2006).

12 Taratula, O., Garbuzenko, O. B., Chen, A. M. & Minko, T. Innovative strategy for treatment of lung cancer: targeted nanotechnology-based inhalation co-delivery of anticancer drugs and siRNA. *J Drug Target* **19**, 900-914, doi:10.3109/1061186X.2011.622404 (2011).

13 Lu, C., Huang, T., Chen, W. & Lu, H. GnRH participates in the self-renewal of A549-derived lung cancer stem-like cells through upregulation of the JNK signaling pathway. *Oncol Rep* **34**, 244-250, doi:10.3892/or.2015.3956 (2015).

14 Limonta, P. *et al.* GnRH receptors in cancer: from cell biology to novel targeted therapeutic strategies. *Endocr Rev* **33**, 784-811, doi:10.1210/er.2012-1014 (2012).

15 Kayacan, O. *et al.* Impact of TNF-alpha and IL-6 levels on development of cachexia in newly diagnosed NSCLC patients. *Am J Clin Oncol* **29**, 328-335, doi:10.1097/01.coc.0000221300.72657.e0 (2006).

16 Yanagawa, H. *et al.* Serum levels of interleukin 6 in patients with lung cancer. *Br J Cancer* **71**, 1095-1098 (1995).

17 Alexandrakis, M. G. *et al.* Evaluation of inflammatory cytokines in malignant and benign pleural effusions. *Oncol Rep* **7**, 1327-1332 (2000).

18 Ujiie, H. *et al.* Serum hepatocyte growth factor and interleukin-6 are effective prognostic markers for non-small cell lung cancer. *Anticancer Res* **32**, 3251-3258 (2012).

19 Lee, J. J. *et al.* A high-affinity protein binder that blocks the IL-6/STAT3 signaling pathway effectively suppresses non-small cell lung cancer. *Mol Ther* **22**, 1254-1265, doi:10.1038/mt.2014.59 (2014).

20 Chen, Y. *et al.* Gprc5a Deletion Enhances the Transformed Phenotype in Normal and Malignant Lung Epithelial Cells by Eliciting Persistent Stat3 Signaling Induced by Autocrine Leukemia Inhibitory Factor. *Cancer Res* **70**, 8917-8926, doi:10.1158/0008-5472.can-10-0518 (2010).

21 Guo, Y., Xu, F., Lu, T., Duan, Z. & Zhang, Z. Interleukin-6 signaling pathway in targeted therapy for cancer. *Cancer Treat Rev* **38**, 904-910, doi:10.1016/j.ctrv.2012.04.007 (2012).

22 Liu, J., Yu, H. & Hu, W. LIF is a new p53 negative regulator. *J Nat Sci* **1**, e131 (2015).

23 Kuphal, S., Wallner, S. & Bosserhoff, A. K. Impact of LIF (leukemia inhibitory factor) expression in malignant melanoma. *Exp Mol Pathol* **95**, 156-165, doi:10.1016/j.yexmp.2013.06.012 (2013).

24 Vicent, S. *et al.* ERK1/2 is activated in non-small-cell lung cancer and associated with advanced tumours. *Br J Cancer* **90**, 1047-1052, doi:10.1038/sj.bjc.6601644 (2004).

25 Chen, X. F. *et al.* Transforming growth factor-beta1 induces epithelial-to-mesenchymal transition in human lung cancer cells via PI3K/Akt and MEK/Erk1/2 signaling pathways. *Mol Biol Rep* **39**, 3549-3556, doi:10.1007/s11033-011-1128-0 (2012).

26 Roberts, P. J. & Der, C. J. Targeting the Raf-MEK-ERK mitogen-activated protein kinase cascade for the treatment of cancer. *Oncogene* **26**, 3291-3310, doi:10.1038/sj.onc.1210422 (2007).

27 Mebratu, Y. & Tesfaigzi, Y. How ERK1/2 Activation Controls Cell Proliferation and Cell Death Is Subcellular Localization the Answer? *Cell cycle (Georgetown, Tex.)* **8**, 1168-1175 (2009).

28 Shi, M., Zheng, Y., Garcia, A., Xu, L. & Foster, D. A. Phospholipase D provides a survival signal in human cancer cells with activated H-Ras or K-Ras. *Cancer Lett* **258**, 268-275, doi:10.1016/j.canlet.2007.09.003 (2007).

29 Imamura, F. *et al.* Induction of in Vitro Tumor Cell Invasion of Cellular Monolayers by Lysophosphatidic Acid or Phospholipase D. *Biochem Biophys Res Commun* **193**, 497-503, doi:10.1006/bbrc.1993.1651 (1993).

30 Bruntz, R. C., Lindsley, C. W. & Brown, H. A. Phospholipase D signaling pathways and phosphatidic acid as therapeutic targets in cancer. *Pharmacol Rev* **66**, 1033-1079, doi:10.1124/pr.114.009217 (2014).

31 Pleasance, E. D. *et al.* A small-cell lung cancer genome with complex signatures of tobacco exposure. *Nature* **463**, 184-190, doi:10.1038/nature08629 (2010).

32 Durak, I. *et al.* Activity of the enzymes participating in purine metabolism of cancerous and noncancerous human kidney tissues. *Cancer Invest* **15**, 212-216 (1997).

33 Weber, G. Enzymes of purine metabolism in cancer. *Clin Biochem* **16**, 57-63 (1983).

34 Supriya Rajanna, A. S. c-Met: A Potential Target for Current Non-Small-Cell Lung Cancer Therapeutics. *Chemother Open Access* **03**, doi:10.4172/2167-7700.1000136 (2014).

35 Ma, P. C. *et al.* Downstream signalling and specific inhibition of c-MET/HGF pathway in small cell lung cancer: implications for tumour invasion. *Br J Cancer* **97**, 368-377, doi:10.1038/sj.bjc.6603884 (2007).

36 Sadiq, A. A. & Salgia, R. MET as a possible target for non-small-cell lung cancer. *J Clin Oncol* **31**, 1089-1096, doi:10.1200/JCO.2012.43.9422 (2013).

37 Landi, L., Minuti, G., D'Incecco, A. & Cappuzzo, F. Targeting c-MET in the battle against advanced nonsmall-cell lung cancer. *Curr Opin Oncol* **25**, 130-136, doi:10.1097/CCO.0b013e32835daf37 (2013).

38 GAO, C. F. & WOUDE, G. F. V. HGF/SF-Met signaling in tumor progression. *Cell Res* **15**, 49-51 (2005).

39 Jung, K. H., Park, B. H. & Hong, S. S. Progress in cancer therapy targeting c-Met signaling pathway. *Arch Pharm Res* **35**, 595-604, doi:10.1007/s12272-012-0402-6 (2012).

40 Gherardi, E., Birchmeier, W., Birchmeier, C. & Vande Woude, G. Targeting MET in cancer: rationale and progress. *Nat Rev Cancer* **12**, 89-103, doi:10.1038/nrc3205 (2012).

41 Liu, X., Yao, W., Newton, R. C. & Scherle, P. A. Targeting the c-MET signaling pathway for cancer therapy. *Expert Opin Investig Drugs* **17**, 997-1011, doi:10.1517/13543784.17.7.997 (2008).

42 Ishida, M. *et al.* Smoking Cessation Reverses DNA Double-Strand Breaks in Human Mononuclear Cells. *PLoS ONE* **9**, e103993, doi:10.1371/journal.pone.0103993 (2014).

43 Paull, T. T. Mechanisms of ATM Activation. *Annu Rev Biochem* **84**, 711-738, doi:10.1146/annurev-biochem-060614-034335 (2015).

44 Nyunoya, T. *et al.* Molecular processes that drive cigarette smoke-induced epithelial cell fate of the lung. *Am J Respir Cell Mol Biol* **50**, 471-482, doi:10.1165/rcmb.2013-0348TR (2014).

45 Jorgensen, E. D., Zhao, H., Traganos, F., Albino, A. P. & Darzynkiewicz, Z. DNA damage response induced by exposure of human lung adenocarcinoma cells to smoke from tobacco-and nicotine-free cigarettes. *Cell Cycle* **9**, 2170-2176 (2010).

46 Cremona, C. A. & Behrens, A. ATM signalling and cancer. *Oncogene* **33**, 3351-3360, doi:10.1038/onc.2013.275 (2014).

47 Li, Y. & Yang, D. Q. The ATM inhibitor KU-55933 suppresses cell proliferation and induces apoptosis by blocking Akt in cancer cells with overactivated Akt. *Mol Cancer Ther* **9**, 113-125, doi:10.1158/1535-7163.mct-08-1189 (2010).

48 Pazolli, E. *et al.* Chromatin remodeling underlies the senescence-associated secretory phenotype of tumor stromal fibroblasts that supports cancer progression. *Cancer Res* **72**, 2251-2261, doi:10.1158/0008-5472.can-11-3386 (2012).

49 He, Y. *et al.* Aberrant expression of proteins involved in signal transduction and DNA repair pathways in lung cancer and their association with clinical parameters. *PLoS One* **7**, e31087, doi:10.1371/journal.pone.0031087 (2012).

50 Ding, L. *et al.* Somatic mutations affect key pathways in lung adenocarcinoma. *Nature* **455**, 1069-1075, doi:10.1038/nature07423 (2008).

51 dos Santos, V. A. *et al.* Glutamate and synaptic plasticity systems and smoking behavior: results from a genetic association study. *PLoS One* **7**, e38666, doi:10.1371/journal.pone.0038666 (2012).

52 D'Souza, M. S. & Markou, A. The "stop" and "go" of nicotine dependence: role of GABA and glutamate. *Cold Spring Harb Perspect Med* **3**, doi:10.1101/cshperspect.a012146 (2013).

53 Rzeski, W., Turski, L. & Ikonomidou, C. Glutamate antagonists limit tumor growth. *Proc Natl Acad Sci U S A* **98**, 6372-6377, doi:10.1073/pnas.091113598 (2001).

54 Stepulak, A. *et al.* NMDA antagonist inhibits the extracellular signal-regulated kinase pathway and suppresses cancer growth. *Proc Natl Acad Sci U S A* **102**, 15605-15610, doi:10.1073/pnas.0507679102 (2005).

55 Stepulak, A., Rola, R., Polberg, K. & Ikonomidou, C. Glutamate and its receptors in cancer. *J Neural Transm (Vienna)* **121**, 933-944, doi:10.1007/s00702-014-1182-6 (2014).

56 Willard, S. S. & Koochekpour, S. Glutamate signaling in benign and malignant disorders: current status, future perspectives, and therapeutic implications. *Int J Biol Sci* **9**, 728-742, doi:10.7150/ijbs.6475 (2013).

57 Choi, K. Y., Chang, K., Pickel, J. M., Badger, J. D., 2nd & Roche, K. W. Expression of the metabotropic glutamate receptor 5 (mGluR5) induces melanoma in transgenic mice. *Proc Natl Acad Sci U S A* **108**, 15219-15224, doi:10.1073/pnas.1107304108 (2011).

58 Robert, J. K. *et al.* in *A23. CHRONIC OBSTRUCTIVE PULMONARY DISEASE: PATHOGENESIS* *American Thoracic Society International Conference Abstracts* A1103-A1103 (American Thoracic Society, 2013).

59 Samanta, D. *et al.* Smoking attenuates transforming growth factor-beta-mediated tumor suppression function through downregulation of Smad3 in lung cancer. *Cancer Prev Res (Phila)* **5**, 453-463, doi:10.1158/1940-6207.CAPR-11-0313 (2012).

60 Checa, M. *et al.* Cigarette Smoke Enhances the Expression of Profibrotic Molecules in Alveolar Epithelial Cells. *PLoS One* **11**, e0150383, doi:10.1371/journal.pone.0150383 (2016).

61 Drabsch, Y. & ten Dijke, P. TGF-β signalling and its role in cancer progression and metastasis. *Cancer Metastasis Rev* **31**, 553-568, doi:10.1007/s10555-012-9375-7 (2012).

62 Huang, A.-L. *et al.* TGF-β1 Protein Expression in Non-Small Cell Lung Cancers is Correlated with Prognosis. *Asian Pac J Cancer Prev* **15**, 8143-8147, doi:10.7314/apjcp.2014.15.19.8143 (2014).

63 Kubiczkova, L., Sedlarikova, L., Hajek, R. & Sevcikova, S. TGF-β–an excellent servant but a bad master. *J Transl Med* **10**, 1 (2012).
